# Ten quick tips for effective dimensionality reduction

**DOI:** 10.1371/journal.pcbi.1006907

**Published:** 2019-06-20

**Authors:** Lan Huong Nguyen, Susan Holmes

**Affiliations:** 1 Institute for Mathematical and Computational Engineering, Stanford University, Stanford, California, United States of America; 2 Department of Statistics, Stanford University, Stanford, California, United States of America; University of Toronto, CANADA

## Introduction

Dimensionality reduction (DR) is frequently applied during the analysis of high-dimensional data. Both a means of denoising and simplification, it can be beneficial for the majority of modern biological datasets, in which it’s not uncommon to have hundreds or even millions of simultaneous measurements collected for a single sample. Because of “the curse of dimensionality,” many statistical methods lack power when applied to high-dimensional data. Even if the number of collected data points is large, they remain sparsely submerged in a voluminous high-dimensional space that is practically impossible to explore exhaustively (see chapter 12 [1]). By reducing the dimensionality of the data, you can often alleviate this challenging and troublesome phenomenon. Low-dimensional data representations that remove noise but retain the signal of interest can be instrumental in understanding hidden structures and patterns. Original high-dimensional data often contain measurements on uninformative or redundant variables. DR can be viewed as a method for latent feature extraction. It is also frequently used for data compression, exploration, and visualization. Although many DR techniques have been developed and implemented in standard data analytic pipelines, they are easy to misuse, and their results are often misinterpreted in practice. This article presents a set of useful guidelines for practitioners specifying how to correctly perform DR, interpret its output, and communicate results. Note that this is not a review article, and we recommend some important reviews in the references.

## Tip 1: Choose an appropriate method

The abundance of available DR methods can seem intimidating when you want to pick one out of the existing bounty for your analysis. The truth is, you don't really need to commit to only one tool; however, you must recognize which methods are appropriate for your application.

The choice of a DR method depends on the nature of your input data. For example, different methods apply to continuous, categorical, count, or distance data. You should also consider your intuition and domain knowledge about the collected measurements. It is often the case that observations can adequately capture only the small-scale relationships between nearby (or similar) data points but not the long-range interactions between distant observations. Considering the nature and the resolution of your data is important, as DR methods can be focused on recovering either global or local structures in the data. In general, linear methods such as principal component analysis (PCA) [2, 3], correspondence analysis (CA) [4], multiple CA (MCA) [5], or classical multidimensional scaling (cMDS), also referred to as principal CA (PCoA) [6], are more adept at preserving global structure, whereas nonlinear methods such as kernel PCA [7, 8], nonmetric multidimensional scaling (NMDS) [9, 10], Isomap [11], diffusion maps [12], and varieties of neighbor embedding (NE) techniques [13] such as t-Stochastic NE (t-SNE) [14] are better at representing local interactions. NE approaches do not preserve long-range interactions between data points and generate visualizations in which the arrangement of nonneighboring groups of observations is not informative. As a consequence, inferences should not be made based on large-scale structures observed in NE plots. Reviews of linear and nonlinear DR methods are provided in [15] and [16], respectively.

If observations in your data have assigned class labels, and your goal is to obtain a representation that best separates them into known categories, you might consider using supervised DR techniques. Examples of supervised DR methods include partial least squares (PLS) [17], linear discriminant analysis (LDA) [18], neighborhood component analysis (NCA) [19], and the bottleneck neural network classifier [20]. Unlike the previously listed unsupervised methods, blind to observations' group memberships, these supervised DR techniques directly use the class assignment information to cluster together data points with the same labels.

For situations in which multidomain data are gathered, e.g., gene expression together with proteomics and methylation data, you might apply DR to each data table separately and then align them using a Procrustes transformation [21] or, instead, consider methods that allow integration of multiple datasets such as the conjoint analysis method for multiple tables known as STATIS [22, 23] and the equivalent method for the conjoint analysis of multiple distance matrices called DiSTATIS [24] (see Tip 9 for more details). Table 1 gives a classification and a summary of the basic properties of the DR techniques. To assist practitioners, we also include in Table 2 a list of stable implementations of methods discussed in this article.

**Table 1:** Dimensionality reduction methods.

Method	Input Data	Method Class	Nonlinear	Complexity
PCA	continuous data	unsupervised		$O(\max(n^{2}p,np^{2}))$
CA	categorical data	unsupervised		$O(\max(n^{2}p,np^{2}))$
MCA	categorical data	unsupervised		$O(\max(n^{2}p,np^{2}))$
PCoA (cMDS)	distance matrix	unsupervised		$O(n^{2}p)$
NMDS	distance matrix	unsupervised		$O(n^{2}h)$
Isomap	continuous*	unsupervised	✔	$O(n^{2}(p+\log\;n))$
Diffusion Map	continuous*	unsupervised	✔	$O(n^{2}p)$
Kernel PCA	continuous*	unsupervised	✔	$O(n^{2}p)$
t-SNE	continuous/distance	unsupervised	✔	$O(n^{2}p+n^{2}h)$
Barnes–Hut t-SNE	continuous/distance	unsupervised	✔	O(nhlogn)
LDA	continuous (X and Y)	supervised		$O(np^{2}+p^{3})$
PLS (NIPALS)	continuous (X and Y)	supervised		O(npd)
NCA	distance matrix	supervised	✔	$O(n^{2}h)$
Bottleneck NN	continuous/categorical	supervised	✔	O(nph)
STATIS	continuous	multidomain		$O(n^{2}P,nP^{2})$
DiSTATIS	distance matrix	multidomain		$O(n^{2}P,nP^{2})$

Basic properties: input data required, method class, linear or nonlinear, and runtime complexity in terms of: *n*—the number of observations, *p*—the number of features in the original data, *k*—the selected number of nearest neighbors, *h*—the number of iterations, and *P*—the total number of variables in all available datasets collected on *n* samples in the case of multidomain data.

*Commonly, Isomap estimates geodesic distances between data points from Euclidean distances, and Diffusion Map and Kernel PCA compute Gaussian kernels and thus require continuous data input. However, it is possible to use categorical data if other dissimilarities or kernels are used.

Abbreviations: CA, correspondence analysis; cMDS, classical multidimensional scaling; LDA, linear discriminant analysis; MCA, multiple CA; NCA, neighborhood component analysis; NIPALS, nonlinear iterative partial least squares; NMDS, nonmetiric multidimensional scaling; NN, neural network; PCA, principal component analysis; PCoA, principal CA; t-SNE, t-Stochastic Neighbor Embedding; PLS, partial least squares

**Table 2:** Example implementations.

Method	R function	Python function
PCA	stats::prcomp	sklearn.decomposition.PCA
CATPCA	gifi::princals	
CA	FactoMineR::CA	
MCA	FactoMineR::MCA	
PCoA (cMDS)	stats::cmdscale	sklearn.manifold.MDS
NMDS	ecodist::nmds	sklearn.manifold.MDS
Isomap	vegan::isomap	sklearn.manifold.Isomap
Diffusion Map	diffusionMap::diffuse	
(Barnes–Hut) t-SNE	Rtsne::Rtsne	sklearn.manifold.TSNE
LDA	MASS::lda	sklearn.discriminant_analysis.LinearDiscriminantAnalysis
PLS (NIPALS)	mixOmics::pls	sklearn.cross_decomposition.PLSRegression
DiSTATIS	DistatisR::distatis	
Procrustes	vegan::procrustes	scipy.spatial.procrustes

Software packages and function performing specified DR techniques available in R and python. R implementations are given as package_name::function_name; listed python functions come from sklearn and scipy libraries. The outputs of most linear DR methods can be visualized in R with factoextra package [25], used to generate a number of the plots in this article. Abbreviations: CA, correspondence analysis; CATPCA, categorical PCA; cMDS, classical multidimensional scaling; DR, dimensionality reduction; LDA, linear discriminant analysis; MCA, multiple CA; NIPALS, nonlinear iterative partial least squares; NMDS, nonmetiric multidimensional scaling; PCA, principal component analysis; PCoA, principal CA; t-SNE, t-Stochastic Neighbor Embedding; PLS, partial least squares

## Tip 2: Preprocess continuous and count input data

Before applying DR, suitable data preprocessing is often necessary. For example, data centering—subtracting variable means from each observation—is a required step for PCA on continuous variables and is applied by default in most standard implementations. Another commonly employed data transformation is scaling—multiplying each measurement of a variable by a scalar factor so that the resulting feature has a variance of one. The scaling step ensures equal contribution from each variable, which is especially important for datasets containing heterogeneous features with highly variable ranges or distinct units, e.g., patient clinical data or environmental factors data.

When the units of all variables are the same, e.g., in high-throughput assays, normalizing feature variances is not advised, because it results in shrinkage of features containing strong signals and inflation of features with no signal. Other data transformations may be required, depending on the application, the type of input data, and the DR method used. For example, if changes in your data are multiplicative, e.g., your variables measure percent increase/decrease, you should consider using a log-transform before applying PCA. When working with genomic sequencing data, two issues need to be addressed before you can apply DR. First, each sequencing sample has a different library size (sequencing depth)—a nuisance parameter that artificially differentiates observations. In order to make observations comparable to each other, samples need to be normalized by dividing each measurement by a corresponding sample size factor, estimated using specialized methods (e.g., DESeq2 [26], edgeR [27]). Secondly, the assay data exhibit a mean-variance trend in which features with higher means have higher variances. A variance stabilization transformation (VST) is needed to adjust for this effect and to avoid bias toward the highly abundant features. For counts with a negative-binomial distribution, such as the sequencing read counts, an inverse hyperbolic sine transformation or similar techniques are recommended [28–30]. Sample normalization and variance stabilization together are effective and sufficient preprocessing steps for high-throughput data.

## Tip 3: Handle categorical input data appropriately

In many cases, available measurements are not numerical but qualitative or categorical. The corresponding data variables represent categories—nonnumeric quantities, e.g., phenotypes, cohort memberships, sample sequencing runs, survey respondent ratings. When the relationship between the levels (distinct values) of two categorical variables is of interest, CA is applied to a contingency table (constructed from the data) whose entries are the categories' co-occurrence frequencies. If more than two categorical variables are available, MCA enables the study of both the relationship between the observations and the associations between variable categories. MCA is a generalization of CA and is simply CA applied to an indicator matrix formed by a dummy (one-hot) encoding of the categorical variables [5]. When the input data contain both numerical and categorical variables, two strategies are available. If only a few categorical variables are present, PCA is used on numerical variables, and the group means for the levels of the categorical variables can be projected as supplementary (unweighted) points (see chapter 9 of [1] for details). On the other hand, if the mixed dataset contains a large number of categorical variables, multiple factor analysis (MFA) [31] can be used. The method applies PCA on numerical and MCA on categorical variables and combines the results by weighing variable groups.

Another approach to working with categorical or mixed data is to perform PCA on variables transformed using an “optimal quantification.” Traditional PCA cannot be applied to categorical variables, because its objective is to maximize the variance accounted for, a concept that exists only for numerical variables. For “nominal” (unordered) or “ordinal” (ordered) categorical variables, variance can be replaced by a chi-squared distance on category frequencies (as in CA), or an appropriate variable transformation can be applied before doing a PCA. Converting categorical variables to dummy binary features is one method; another approach is to use optimal scaling categorical PCA (CATPCA) [32–34]. Optimal scaling replaces original levels of categorical variables with category quantifications such that the variance in the new variables is maximized [35]. CATPCA is then formulated as an optimization problem, in which the squared difference between the quantified data and the principal component is minimized iteratively, alternating between the component scores, the component loadings, and the variable quantification.

An advantage of optimal scaling is that it does not assume a linear relationship between variables. In fact, the ability of CATPCA to handle nonlinear relations between variables is important even when the input data are all numeric. Thus, when nonlinearities are present and the standard PCA explains only a low proportion of the variance, optimal scaling provides a possible remedy.

## Tip 4: Use embedding methods for reducing similarity and dissimilarity input data

When neither quantitative nor qualitative features are available, the relationships between data points, measured as dissimilarities (or similarities), can be the basis of DR performed as a low-dimensional embedding. Even when variable measurements are available, computing dissimilarities and using distance-based methods might be an effective approach. Make sure that you choose a dissimilarity metric that provides the best summary of your data, e.g., if the original data are binary, the Euclidean distance is not appropriate, and the Manhattan distance is better. If the features are sparse, however, then the Jaccard distance is preferred.

cMDS/PCoA and NMDS use pairwise dissimilarities between data points to find an embedding in Euclidean space that provides the best approximation to the supplied distances. Whereas cMDS is a matrix decomposition method akin to PCA, NMDS is an optimization technique that strives to retain only the ordering of the dissimilarities [36]. The latter approach is more applicable when you have low confidence in the values of the input distances. When the dissimilarity data are only available in nonstandard, qualitative formats, more specialized ordinal embedding methods are available, discussed in detail by Kleindessner and von Luxburg in [37, 38]. When using optimization-based multidimensional scaling (MDS), you can choose to preserve only the local interactions by restricting the minimization problem to only the distances from data points to their neighbors, e.g., the k-nearest neighbors. This approach can be referred to as “local” MDS.

Dissimilarities can also be used as input to t-SNE. Similar to local MDS, t-SNE is only focused on representing the short-range interactions. However, the method achieves locality in a different way, by converting the supplied distances into proximity measures using a small-tail Gaussian kernel. A collection of neural network–based approaches, called word2vec [39], have been developed that also use similarity data (the co-occurrence data) to generate vector embeddings of objects in a continuous Euclidean space. These techniques have proven highly effective at generating word embeddings from text corpus–derived data and have since been adapted for gene coexpression data in the gene2vec program by Du and colleagues [40]. The robustness of these highly computational methods has not been yet extensively tested on many biological datasets.

## Tip 5: Consciously decide on the number of dimensions to retain

When performing DR, choosing a suitable number of new dimensions to compute is crucial. This step determines whether the signal of interest is captured in the reduced data, especially important when DR is applied as a preprocessing step preceding statistical analyses or machine learning tasks (e.g., clustering). Even when your primary goal is data visualization, in which only two or three axes can be displayed at a time, you still need to select a sufficient number of new features to generate. For example, the first two or three PCs might explain an insufficient fraction of the variance, in which case the higher-order components should be retained, and multiple combinations of the components should be used for visualizations (e.g., PC1 versus PC2, PC2 versus PC4, and PC3 versus PC5 etc.) In some cases, the strongest signal is a confounding factor, and the variation of interest is captured by higher-order PCs. If this is the case, you must use higher-order components to expose the desired patterns.

**Fig 1:**
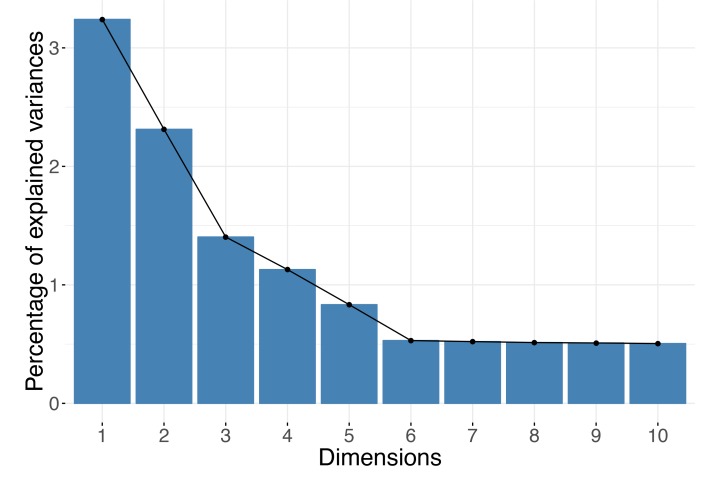
Scree plot. For spectral methods, the eigenvalues can be used to decide how many dimensions are sufficient. The number of dimensions to keep can be selected based on an "elbow rule." In the example shown, you should keep the first five principal components.

The optimal choice for the number of dimensions to keep depends largely on the data itself. You cannot decide on the right dimension for the output before consulting the data. Remember that the number of dimensions can be at most the minimum of the number of observations (rows) and the number of variables (columns) in your dataset. For example, if your dataset contains expression of 10,000 genes but for only 10 samples, there could not be more than 10 (or even 9 if the input data have been centered) axes in your reduced data representation. For DR methods based on spectral decompositions, such as PCA or PCoA, you could use the distribution of the eigenvalues to guide your choice of dimensions. In practice, people usually rely on “scree plots” (example in Fig 1) and “the elbow rule” when making decisions. A scree plot simply shows the eigenvalues corresponding to each of the axes in the output representation or, equivalently, the proportion of the variance each axis (e.g., a PC) explains. When viewing the plot, you should look for a cutoff point, in which an eigenvalue drops significantly below the level of the one immediately preceding it—the "elbow" point. Alternatively, you can inspect a histogram of the eigenvalues and search for the large values that "stand out" from the bulk. Formally, the Marchenko–Pastur distribution asymptotically models the distribution of the singular values of large random matrices. Therefore, for datasets large in both the number of observations and features, you use a rule of retaining only eigenvalues outside the support of the fitted Marchenko–Pastur distribution; however, remember that this applies only when your data have at least thousands of samples and thousands of features.

For nonspectral, optimization-based methods, the number of components is usually prespecified before DR computations. When using these approaches, the number of components can be chosen by repeating the DR process using an increasing number of dimensions and evaluating whether incorporating more components achieves a significantly lower value of the loss function that the method minimizes, e.g., the Kullback–Leibler (KL) divergence between transition probabilities defined for the input and the output data in the case of t-SNE. Ideally, you would like your findings (e.g., patterns seen in visualizations) to be robust to the number of dimensions you choose.

## Tip 6: Apply the correct aspect ratio for your visualizations

Visualization is an important part of the data exploration process. Therefore, it is crucial that the DR plots you generate accurately reflect the output of the DR methods you use. An important but frequently overlooked attribute of a visualization is its aspect ratio. The proportional relationship between the height and the width (and also the depth) of a 2D (and 3D) plot can strongly influence your perception of the data; therefore, the DR plots should obey the aspect ratio consistent with the relative amount of information explained by the output axes displayed.

In the case of PCA or PCoA, each output dimension has a corresponding eigenvalue proportional to the amount of variance it explains. If the relationship between the height and the width of a plot is arbitrary, an adequate picture of the data cannot be attained. Two-dimensional PCA plots with equal height and width are misleading but frequently encountered because popular software programs for analyzing biological data often produce square (2D) or cubical (3D) graphics by default. Instead, the height-to-width ratio of a PCA plot should be consistent with the ratio between the corresponding eigenvalues. Because eigenvalues reflect the variance in coordinates of the associated PCs, you only need to ensure that in the plots, one "unit" in direction of one PC has the same length as one "unit" in direction of another PC. (If you use ggplot2 R package for generating plots, adding + coords_fixed(1) will ensure a correct aspect ratio.)

**Fig 2:**
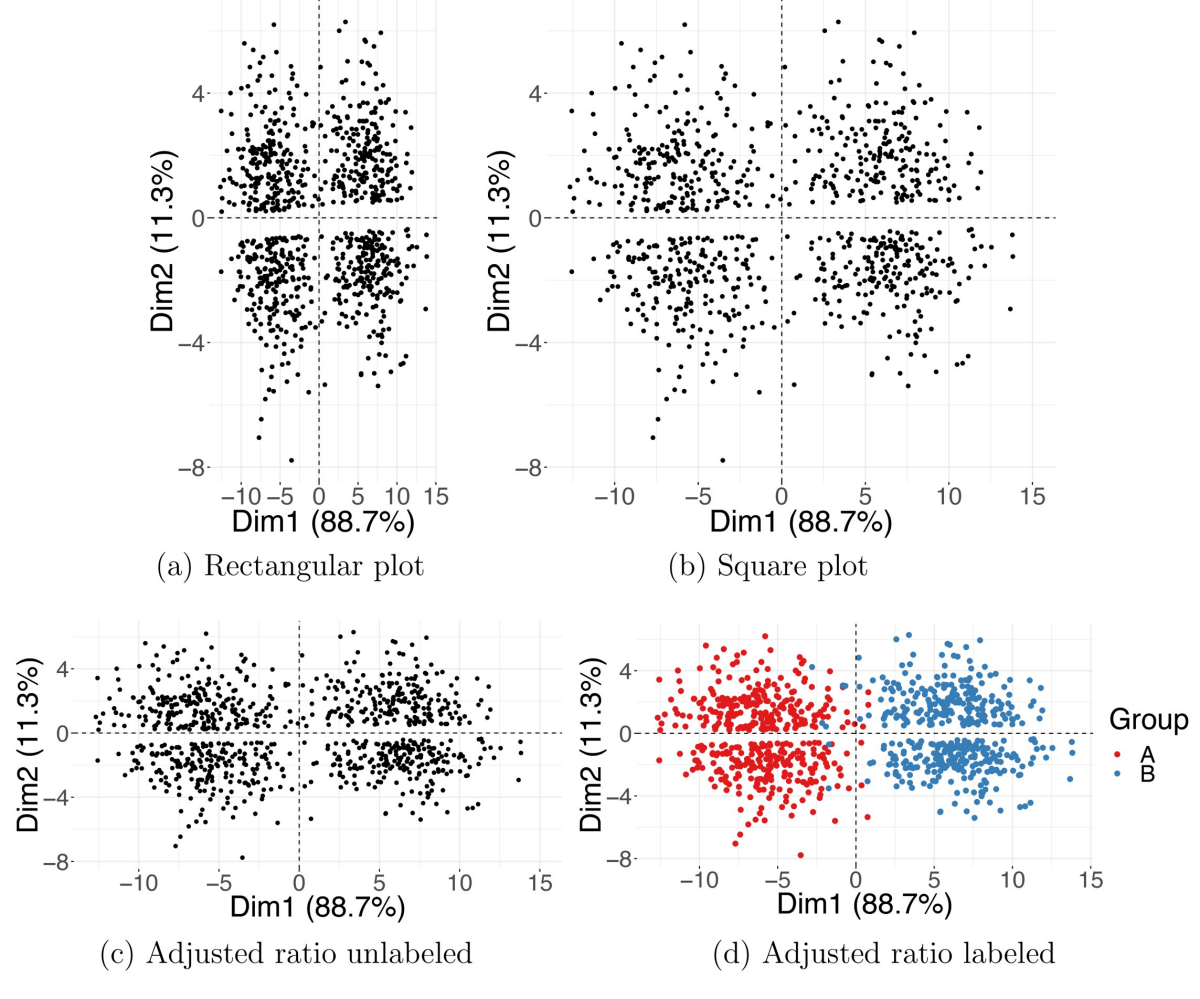
Aspect ratio for PCA plots. Two simulated Gaussian clusters projected on the first and the second PCs. Incorrect aspect ratio in a rectangular (a) and square (b) plot. Correct aspect ratio in (c, d) where the plot's height and width are adjusted to match the variances in PC1 and PC2 coordinates. Colors shown in (d) indicate the true Gaussian group membership. Dim1, dimension 1; Dim2, dimension 2; PC, principal component; PCA, PC analysis.

The aspect ratio issue is illustrated with a simulated example, depicted in Fig 2. In the rectangular (Fig 2A) and the square (Fig 2B) plots, the aspect ratio is inconsistent with the variance of the PC1 and PC2 coordinates; the result is an (incorrect) apparent grouping of the data points into a top and a bottom cluster. In contrast, Fig 2C, with lengths of the two axes set to respect the ratio between the corresponding eigenvalues, shows correct clustering, consistent with the true class assignment. For more examples of how the aspect ratio can affect the plot interpretation, see chapters 7 and 9 of [1].

The ordering of the dimensions is not meaningful in many optimization-based DR methods. For example, in the case of t-SNE, you can choose the number of output dimensions (usually two or three) before computing the new representation. Unlike the PCs, the t-SNE dimensions are unordered and equally important because they have the same weight in the loss function minimized by the optimization algorithm. Thus, for t-SNE, the convention is to make the projection plots square or cubical.

## Tip 7: Understand the meaning of the new dimensions

Many linear DR methods, including PCA and CA, provide a reduced representation both for the observations and for the variables. Feature maps or correlation circles can be used to determine which original variables are associated with each other or with the newly generated output dimensions. The angles between the feature vectors or with the PC axes are informative: vectors at approximately 0° (180°) with each other indicate that the corresponding variables are closely, positively (negatively) related, whereas vectors with a 90° angle indicate rough independence.

**Fig 3:**
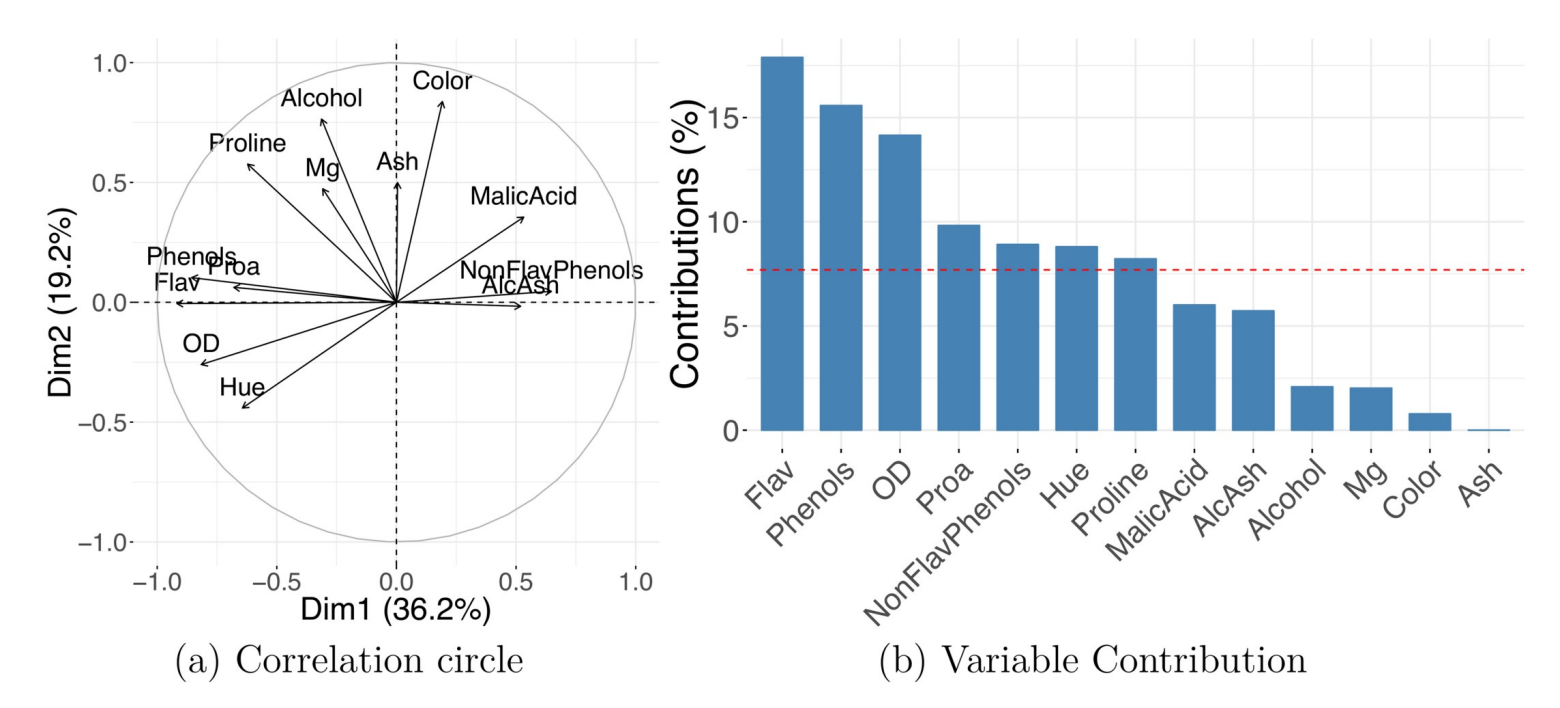
Variables' projection. PCA on wine dataset shows how variables' representation can be used to understand the meaning of the new dimensions. Correlation circle (a) and PC1 contribution plot (b). AlcAsh, alcalinity of ash; Dim1, dimension 1; Dim2, dimension 2; Flav, flavanoids; NonFlav Phenols, nonflavanoid phenols; OD, OD280/OD315 of diluted wine; PC, principal component; PCA, PC analysis; Phenols, total phenols; Proa, proanthocyanins.

Fig 3A shows a correlation circle with scaled coordinates of the variables' projection. The plot indicates that high values of PC1 indicate low values in "Flav" (flavanoids) and "Phenols" (total phenols) and high values in "Malic Acid" and "AlcAsh"(alcalinity of ash). Additionally, "AlcAsh" (alcalinity of ash) levels seem to be closely negatively correlated with "NonFlav Phenols" (nonflavanoid phenols) and independent of "Alcohol" levels.

Original variables' importances to the new dimensions can be visualized using contribution bar plots. A variable's contribution to a given new axis is computed as the ratio between its squared coordinate (in this axis) and the corresponding sum over all variables; the ratio is often converted to percentages. Many programs provide the variables' contributions as standard output; these contributions can be defined for not only a single but also multiple DR axes by summing the values corresponding to selected components. Fig 3B shows variables' percent contribution to PC1; note that the percent contribution does not carry information on the direction of the correlation. When working with high-dimensional datasets such as high-throughput assays, a contribution bar plot for thousands or more variables is not practical; instead, you can limit the plot, showing only the top few (e.g., 20) features with highest contribution.

**Fig 4:**
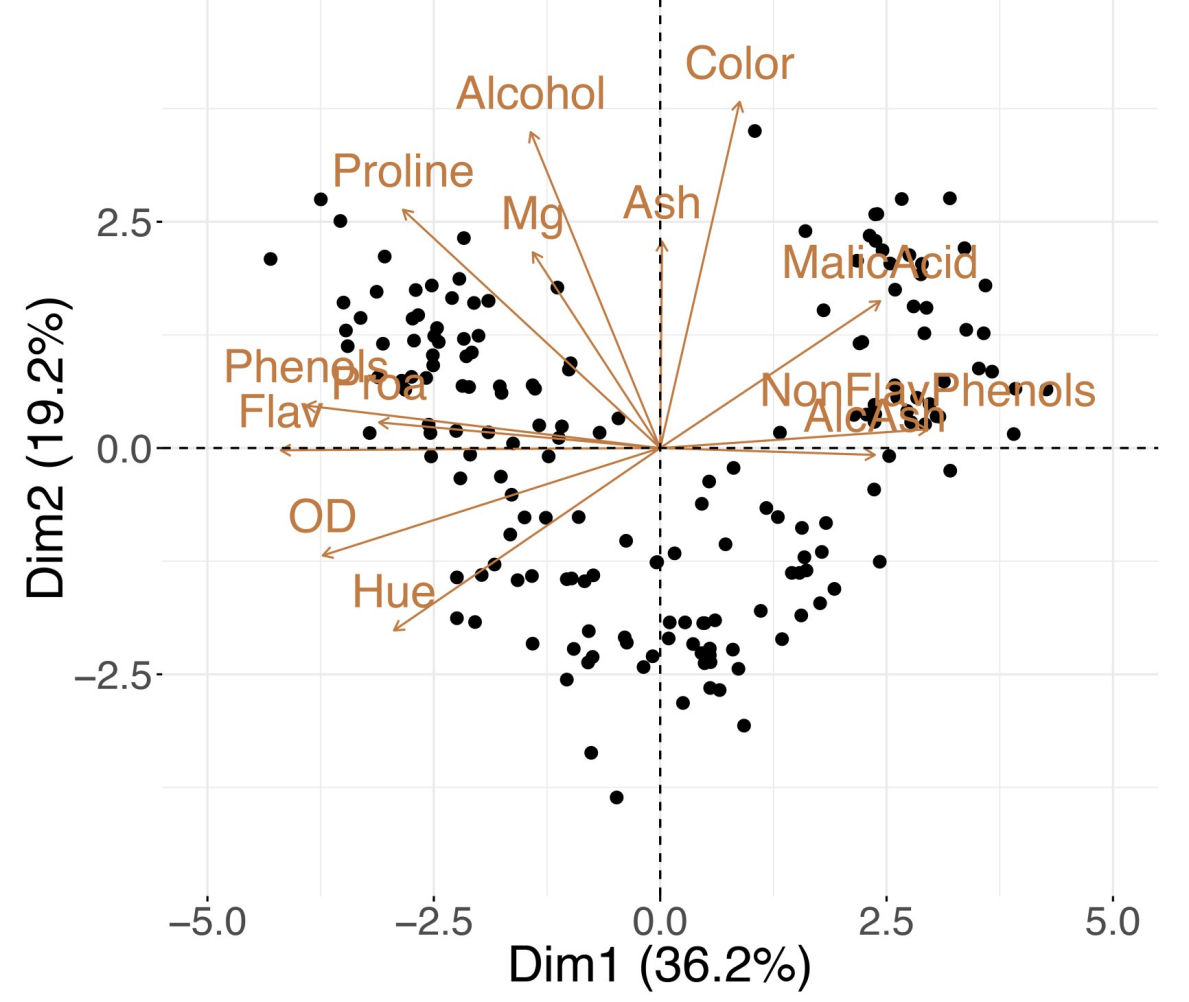
PCA biplot. A single plot for the wine dataset combines both the samples' and the variables' projection to the first two principal components. AlcAsh, alcalinity of ash; Dim1, dimension 1; Dim2, dimension 2; Flav, flavanoids; NonFlav Phenols, nonflavanoid phenols; OD, OD280/OD315 of diluted wine; PCA, principal component analysis; Phenols, total phenols; Proa, proanthocyanins.

Variables and observations can be included in the same graphic—referred to as a “biplot.” The term was coined by Kuno Ruben Gabriel [41] in 1971, but similar ideas were proposed by Jolicoeur and Mosimann already in 1960 [42]. Biplots such as the one in Fig 4 allow you to explore the trends in the data samples and features simultaneously; looking at both at the same time, you might discover groups of similar (close by) observations that have high or low values for certain measured variables (see Tip 8 for further details).

## Tip 8: Find the hidden signal

The primary objective of DR is to compress data while preserving most of the meaningful information. Compression facilitates the process of understanding the data because the reduced data representation is expected to capture the dominant sources of variation more efficiently. The aim is to uncover the "hidden variables" that can successfully expose the underlying structure of the data. The most frequently encountered latent patterns are discrete clusters or continuous gradients.

**Fig 5:**
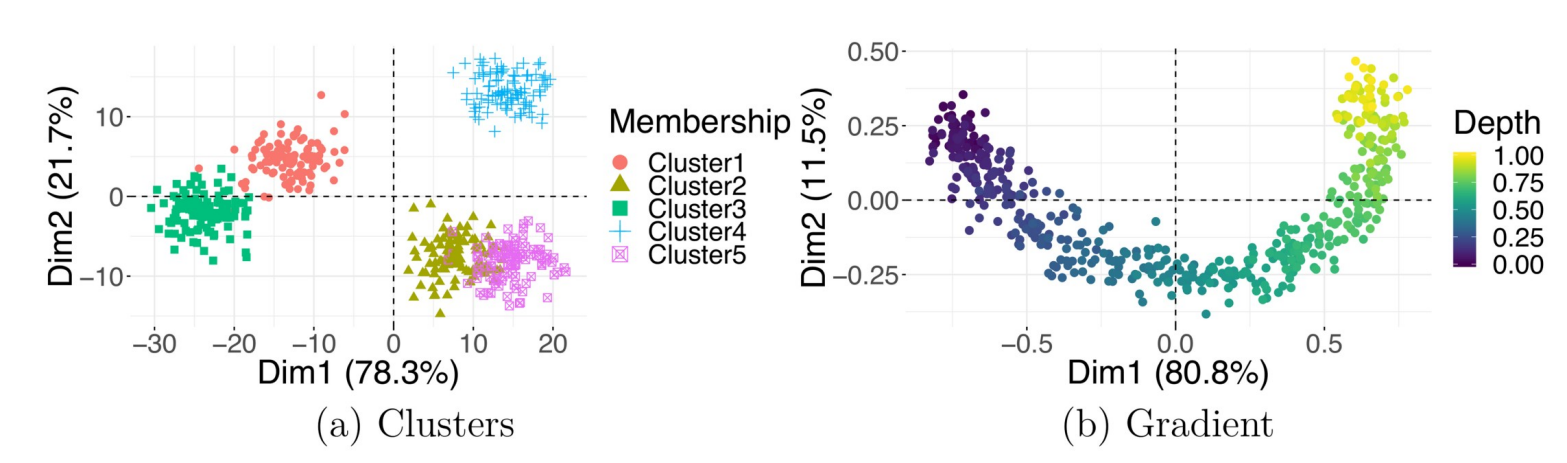
Latent structure. Observations in PCA plots may cluster into groups (a) or follow a continuous gradient (b). Dim1, dimension 1; Dim2, dimension 2; PCA, principal component analysis.

In the former case, similar observations bundle together away from other groups. An example of a simulated clustered dataset is shown in Fig 5A. When performing the cluster analysis, in which the goal is to estimate samples' group memberships, it is common practice to first apply PCA. More specifically, practitioners often use a set of the top (e.g., 50) PCs as input to a clustering algorithm. PCA reduction is intended as a noise-reduction step because the top eigenvectors are expected to contain all signals of interest [43]. Regrettably, this property does not extend to all DR methods. The output generated by neighborhood embedding techniques, such as t-SNE, should not be used for clustering, as they preserve neither distances nor densities—both quantities highly important in the interpretation of clustering output.

Unlike discrete clusters, continuous changes in the data are less frequently identified. It is important to know how to identify and accurately interpret latent gradients, as they often appear in biological data associated with unknown continuous processes. Gradients are present when data points do not separate into distinct tightly packed clusters but instead exhibit a gradual shift from one extreme to another; they often emerge as smooth curves in DR visualizations. It is worth noting that data points are often arranged in horseshoes or arch-shaped configurations when PCA and cMDS (PCoA) is applied to data involving a linear gradient. A “horseshoe effect” can appear in PCA and cMDS plots when the associated eigenvectors take on a specific form [44] because of the properties of the data covariance or distance matrices used for computations, in particular, when these matrices can be expressed as centrosymmetric Kac–Murdock–Szego matrices [45].

You can see an example of this pattern for simulated data with a latent gradient in Fig 5B. Continuous transitions are frequently encountered when measurements are taken over time; for example, the cell development literature is rich with publications introducing methods for analyzing pseudotime, a gradient observed during cell differentiation or development [46, 47]. There can be multiple gradients affecting the data, and a steady change can be recorded in different directions [48]. However, the variable behind the observed continuous gradient could be unknown. In this case, you should focus on finding the discrepancies between the observations at the endpoints (extremes) of the gradients by inspecting the differences between their values for any available external covariates [49], if collected (see Tip 7). Otherwise, you might need to gather additional information on the samples in your dataset to investigate the explanation of these differences.

**Fig 6:**
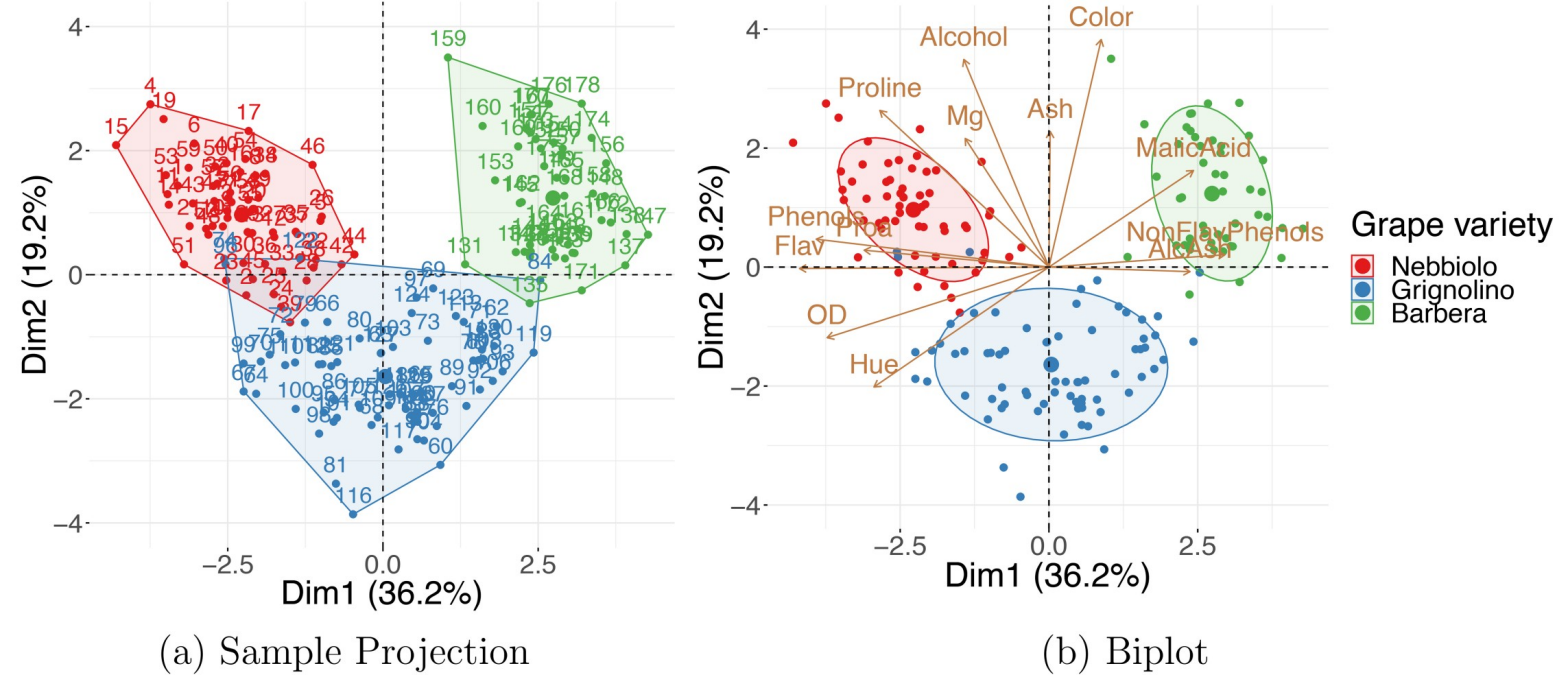
Using external information. (a) A PCA sample projection on the wine dataset shows that, based on their properties, wines tend to cluster in agreement with the grape variety classification: Nebbiolo, Grignolino, and Barbera. (b) A PCA biplot can be used to find which groups of wines tend to have higher levels of which property. Dim1, dimension 1; Dim2, dimension 2; PCA, principal component analysis.

Additional contiguous measurements—those not used for DR computations—are frequently collected on observations included in the dataset. The extra information can be used to improve the understanding of the data. The simplest and most common way to use the external covariates is to include them in DR visualizations—with their values encoded as color, shape, size, or even transparency of corresponding points on the plot. An example of this is shown in Fig 6A: the PCA embedding for a dataset on wine properties [50], in which the data points are colored by wine class, a variable that the DR was blind to. The observed grouping of the wines suggests that 13 wine properties used for DR can characterize the wine categories well. The "Wine Data Set" is accessible from the University of California Irvine (UCI) Machine Learning Repository [51].

Sometimes, directly plotting the external variable against the newly computed features is an effective way of exposing trends present in the data. For example, a scatter plot of a continuous variable, e.g., a patient's age or weight, versus coordinates of a selected output dimension shows correlation between the selected covariate and the new feature. If the external information is categorical instead of continuous, a boxplot of the PC coordinates (e.g., PC1, PC2, or others) can be generated for each level of the variable.

External information can also be incorporated in biplots. Fig 6B shows how combining the external information on the observations with the interpretation of the new axes in terms of the original variables (as described in Tip 7) allows you to discover that “Barbera” wines tend to have higher values of "Malic Acid" and lower "Flavanoids," and “Grignolinos” tend to have low "Ash" and "Alcohol" content.

Additionally, external information can be used to discover batch effects. Batch effects are technical or systemic sources of variation that obscure the main signal of interest. They are frequently encountered in sequencing data, in which samples from the same sequencing run (lane) cluster close together. Because batch effects can confound the signal of interest, it is a good practice to check for their presence and, if found, to remove them before proceeding with further downstream analysis. You can detect technical or systemic variations by generating a DR embedding map with the data points colored by their batch membership, e.g., by the sequencing run, the cage number, the study cohort. If a batch effect is discovered, you can remove it by shifting all observations in such a way that each batch has its centroid (the group's barycenter) located at the center of the plot (usually the origin of the coordinate system).

## Tip 9: Take advantage of multidomain data

Sometimes, more than one set of measurements is collected for the same set of samples; for example, high-throughput genomic studies involving data from multiple domains are often encountered. For the same biological sample microarray gene expression, miRNA expression, proteomics, and DNA methylation data might be gathered [52]. Integrating multiple datasets allows you to both obtain a more accurate representation of higher-order interactions and evaluate the associated variability. Samples often exhibit varying levels of uncertainty, as different regions of the data can be subject to different rates of changes or fluctuations.

One way of dealing with “multidomain,” also referred to as “multimodal,” “multiway,” “multiview,” or “multiomics” data, is to perform DR for each dataset separately and then align them together using a Procrustes transformation—a combination of translation, scaling, and rotation to align one configuration with another as closely as possible (see [21] and [36]). A number of more advanced methods have also been developed, for instance, STATIS [22] and DiSTATIS [24, 53] are generalizations of PCA and classical MDS, respectively. Both methods are used to analyze several sets of data tables collected on the same set of observations, and both are based on an idea of combining datasets into a common consensus structure called the “compromise” [54].

**Fig 7:**
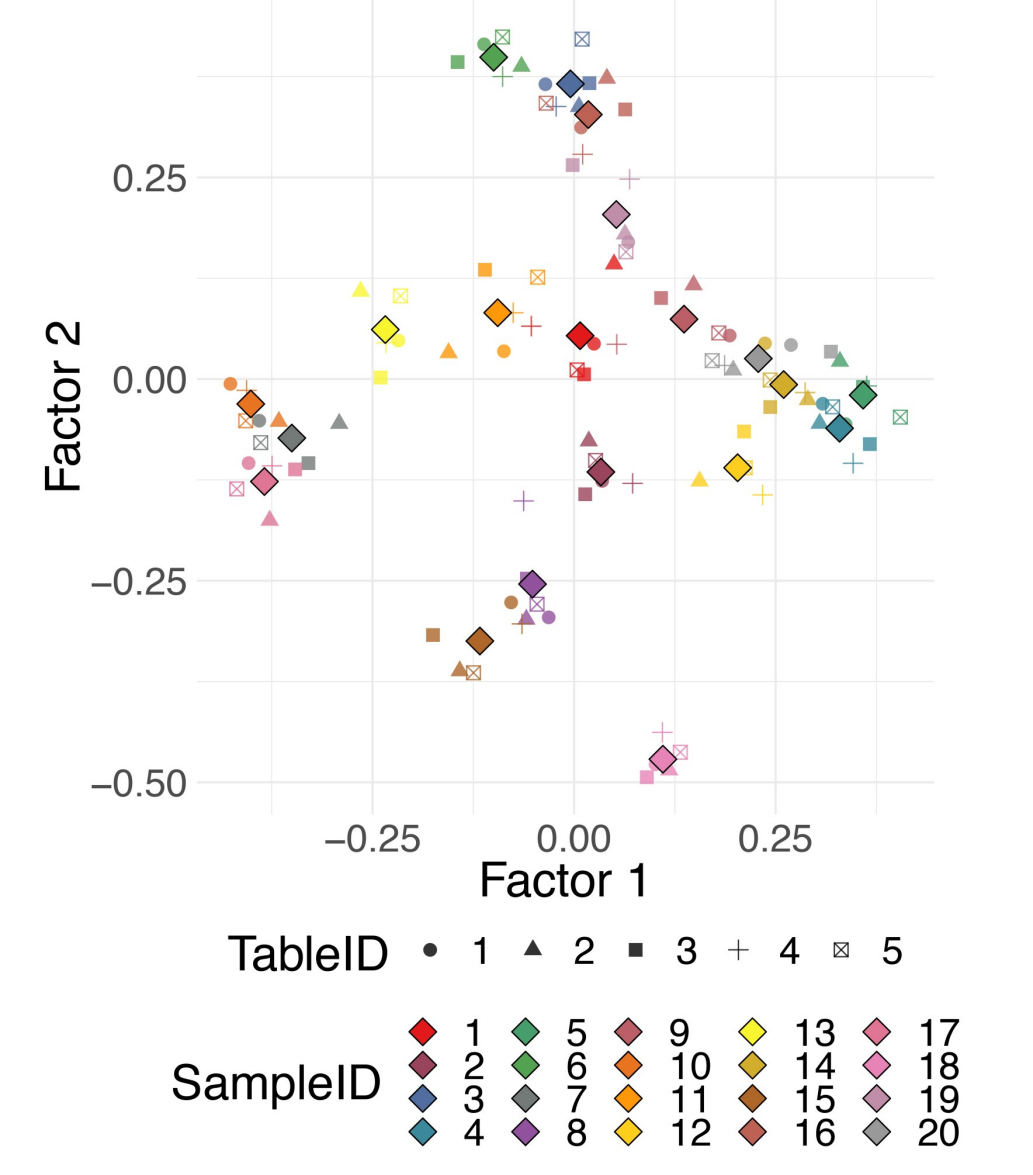
Multidomain data. DiSTATIS on multiple distance tables defined for the same observations. Multiple distances can be computed from different data modalities, e.g., gene expression, methylation, clinical data, or from data resampled from a known data-generating distribution.

The datasets can all be projected onto this consensus space. The projections of individual datasets can be helpful for observing different patterns in observations characterized by data from different domains. Fig 7 shows an example of the use of DiSTATIS on five simulated distance tables for 20 synthetic data points. Different colors correspond to different data points, and different shapes correspond to different distance tables. The compromise points between the tables are denoted with larger diamond shape markers. For a detailed survey on the analysis of multitable data, with a focus on biological multiomics datasets, see Meng and colleagues [55].

## Tip 10: Check the robustness of your results and quantify uncertainties

**Fig 8:**
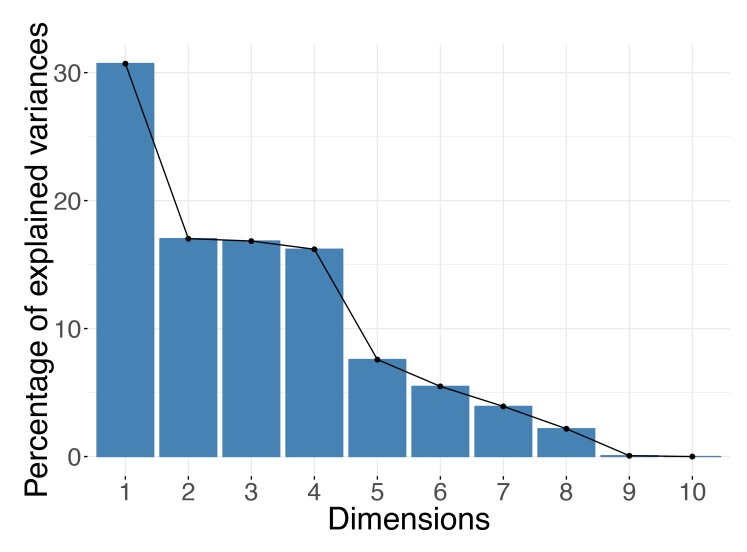
Unstable eigenvalues. When subsequent eigenvalues have close-to-equal values, PCA representation is unstable. PCA, principal component analysis.

For some datasets, the PCA PCs are ill defined, i.e., two or more successive PCs may have very similar variances, and the corresponding eigenvalues are almost exactly the same, as in Fig 8. Although a subspace spanned by these components together is meaningful, the eigenvectors (PCs) are not informative individually, and their loadings cannot be interpreted separately, because a very slight change in even one observation can lead to a completely different set of eigenvectors [1]. In these cases, we say that these PCs are unstable. The dimensions corresponding to similar eigenvalues need to be kept together and not individually interpreted.

When working with techniques that require parameter specification, you should also check the stability of your results against different parameter settings. For example, when running t-SNE, you need to pick a value for perplexity, and different settings can alter the results obtained even qualitatively. It has been frequently observed that when the perplexity is set to a very small value, “artificial clusters” start forming in t-SNE plots [56]. You should not use the values of the t-SNE objective function, the KL divergence, as a criterion to choose an "optimal perplexity," because the KL divergence always decreases (monotonically) as perplexity values increase. For t-SNE, a Bayesian information criterion (BIC)-type rule for selecting perplexities was proposed by Cao and Wang in [57]. However, in practice, you should repeat DR computations for a range of input parameters and visually evaluate whether the patterns discovered are consistent across varying specifications, as stability theory for t-SNE has not yet been developed. In particular, if the clustering pattern disappears with only a slight increase of the perplexity value, the grouping you observed might be only an artifact due to an unsuitably small choice of the parameter.

A separate concern is a method's stability against outliers. In general, it is known that observations far from the origin have more influence on the PCs than the ones close to the center; sometimes it is possible that only a small fraction of the samples in the data almost fully determines the PCs. You should be mindful of these situations and verify that the structure captured by DR represents the bulk of the data and not just a few outliers. In DR maps, the outliers are the remote points, distant from the majority of the observations. In the case of PCA and other linear methods, if all of the points in a sample projection plot are located close to the origin (the center of the plot), with only one or a few points lying very far away, the DR solution is said to be dominated by the outliers. You should inspect suitable data-specific quality control metrics for these points and consider their removal. If samples are removed, the DR needs to be recomputed, and the changes in the output representation should be noted. Observe how observations shift by comparing the DR visualizations before and after the removal of the outliers. You should consider removing not only the technical outliers but also the "outgroups," the aberrant groups known to be extensively different from the majority of the data. Eliminating the outgroups and recomputing the DR allows for patterns in the bulk of the data to emerge. On the other hand, if a dataset contains many aberrant observations, stable methods such as robust kernel PCA [58] should be used.

**Fig 9:**
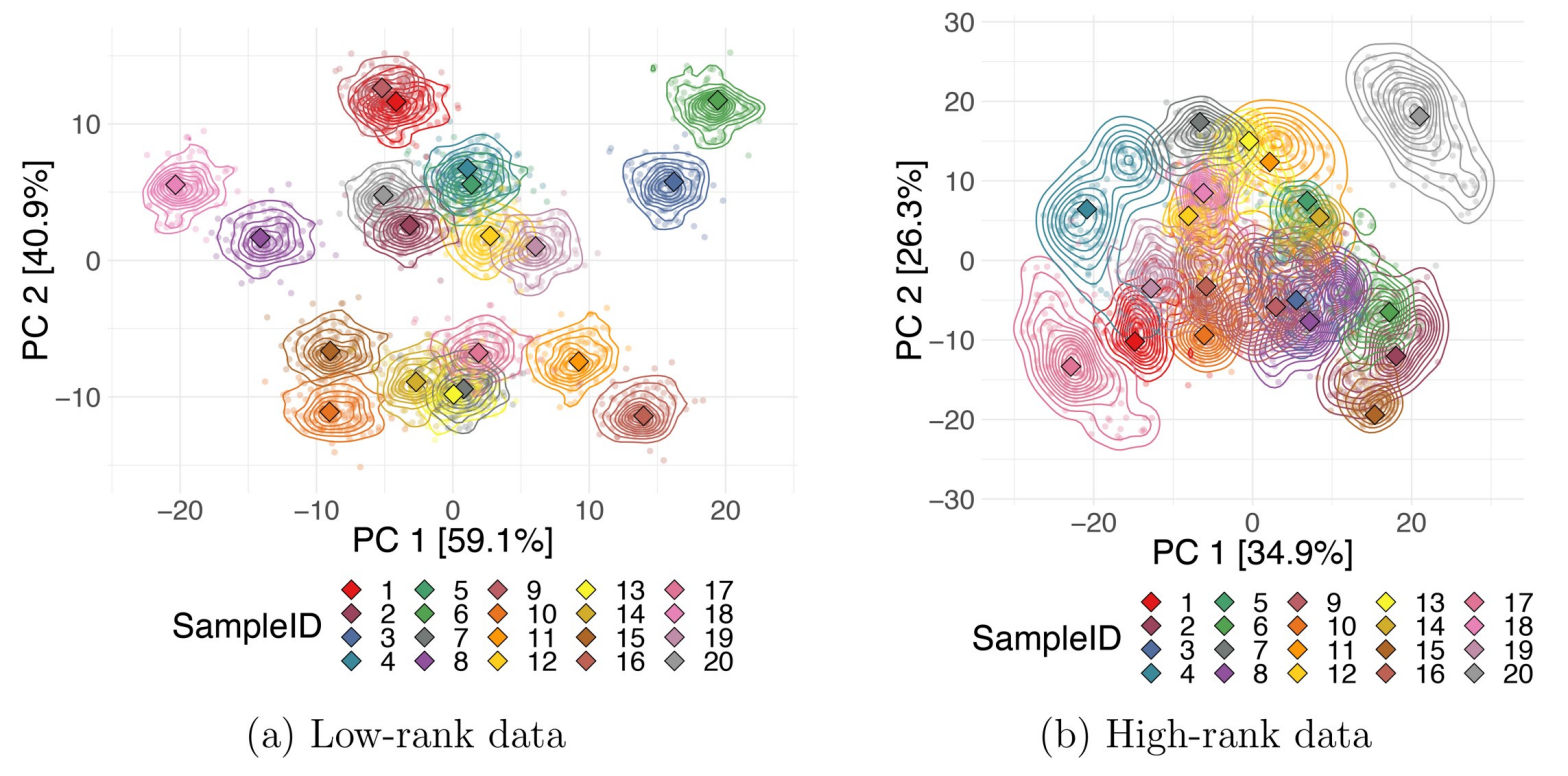
Data point uncertainties. Stability in the DR output coordinates for each data point. Projections of bootstrap samples for two 10D simulated datasets with rank 2 (a) and rank 5 (b) onto the first two PCs aligned using a Procrustes transformation. Smaller, circular markers correspond to each bootstrap trial, and larger, diamond markers are coordinates of the full dataset. DR, dimensionality reduction; PC, principal component.

Additionally, you can estimate the uncertainties associated with observations by constructing a collection of "bootstrap" datasets, i.e., random subsets of the data generated by resampling observations with replacement. The bootstrap set can be treated as multiway data, and the STATIS or Procrustes aligning method described in Tip 8 can be applied to "match" the random subsets together. When a realistic noise model for the data is available, instead of using bootstrap subsamples, you can generate copies of all data points by perturbing the measurement values for each sample and then applying the STATIS or DiSTATIS methods as described in the previous tip to generate the coordinates for the “compromise” and for each individual perturbed copy of the data. Obtaining multiple coordinates estimates per data point allows you to estimate the corresponding uncertainty. You can visualize each sample's uncertainty on a DR embedding map using density contours or by plotting all data points from each bootstrap's projection onto the compromise. Fig 9 shows the Procrustes alignments of PCA projections for two simulated datasets. The colored lines indicate density contours for the output coordinates of the bootstrap subsets, and the diamond markers correspond to the coordinates of the projection of the full data. Plots were produced for 20 synthetic data points from a true 2D and 5D Gaussian, both orthogonally projected to 10 dimensions. We can observe that uncertainties for points in the lower-rank data are much smaller, i.e., the first 2 PCs represent the first dataset better than the second one.

## Conclusion

DR is very useful and sometimes essential when analyzing high-dimensional data. Despite their widespread adoption, DR methods are often misused or misinterpreted. Researchers performing DR might find the sheer number of available methods already intimidating, not to mention the wide variety of different dissimilarity metrics or parameter settings required by some of these methods. This set of ten tips serves as a checklist or informal guideline for practitioners. We described a general step-by-step procedure for performing effective DR and gave pointers for correctly interpreting and adequately communicating the output of DR algorithms. Most of the recommendations discussed here apply to any DR method, but some were instructions directed toward specific reduction approaches.

In addition to everything discussed earlier, we would like to offer one extra piece of advice: keep track of all the decisions you make, including the method you select, the distances or kernels you choose, and the values of parameters you use. The most convenient way to save all steps of your work together with the results obtained is through an R, an IPython, or a Jupyter notebook; these applications allow you to generate a full analysis report containing narrative text, code, and its output. Recording your choices is a crucial part of reproducible research [59]; it allows others to replicate the same results you obtained and speeds up your analysis process the next time you work with similar data. We provide an example of a reproducible report generated with R-markdown in the S1 Text and S1 Code files.

## Supporting information

S1 Code An R-markdown file containing a reproducible record of all plots included in this article. (RMD)

S1 Text A pdf report rendered from an R-markdown file in S1 Code containing text, figures, and code. (PDF)

## References

[1] Holmes S, Huber W. Modern Statistics for Modern Biology. Cambridge, UK: Cambridge University Press; 2019 [cited 2019 May 30]. Available from: https://www.huber.embl.de/msmb/.

[2] Pearson K. On lines and planes of closest fit to systems of points in space. The London, Edinburgh, and Dublin Philosophical Magazine and Journal of Science. 1901;2(11):559–572. 10.1080/14786440109462720

[3] Hotelling H. Analysis of a Complex of Statistical Variables with Principal Components. Journal of Educational Psychology. 1933;24:417–441.

[4] Hirschfeld HO. A Connection between Correlation and Contingency. Mathematical Proceedings of the Cambridge Philosophical Society. 1935;31(4):520–524. 10.1017/S0305004100013517

[5] Abdi H, Valentin D. Multiple Correspondence Analysis. Encyclopedia of Measurement and Statistics. 2007 10.4135/9781412952644

[6] Torgerson WS. Theory and methods of scaling. Oxford, UK: Wiley; 1958.

[7] Schölkopf B, Smola A, Müller KR. Nonlinear Component Analysis as a Kernel Eigenvalue Problem. Neural Computation. 1998;10(5):1299–1319. 10.1162/089976698300017467

[8] Schölkopf B, Smola AJ, Müller KR. Advances in Kernel Methods. Cambridge, MA: MIT Press; 1999 p. 327–352.

[9] Shepard RN. The analysis of proximities: Multidimensional scaling with an unknown distance function. II. Psychometrika. 1962;27(3):219–246. 10.1007/BF02289621

[10] Kruskal JB. Nonmetric multidimensional scaling: A numerical method. Psychometrika. 1964;29(2):115–129. 10.1007/BF02289694

[11] Tenenbaum JB, Silva Vd, Langford JC. A Global Geometric Framework for Nonlinear Dimensionality Reduction. Science. 2000;290(5500):2319–2323. 10.1126/science.290.5500.2319 11125149

[12] Coifman RR, Lafon S. Diffusion maps. Applied and Computational Harmonic Analysis. 2006;21(1):5–30. 10.1016/j.acha.2006.04.006

[13] Hinton GE, Roweis ST. Stochastic Neighbor Embedding. In: Becker S, Thrun S, Obermayer K, editors. Advances in Neural Information Processing Systems 15. Proceedings of the 2002 Neural Information processing Systems Conference. Cambridge, MA: MIT Press; 2003. p. 857–864.

[14] van der Maaten LJP, Hinton G. Visualizing Data using t-SNE. Journal of Machine Learning Research. 2008;9:2579–2605.

[15] Cunningham JP, Ghahramani Z. Linear Dimensionality Reduction: Survey, Insights, and Generalizations. Journal of Machine Learning Research. 2015;16:2859–2900.

[16] Ting D, Jordan MI. On Nonlinear Dimensionality Reduction, Linear Smoothing and Autoencoding. arXiv:1803.02432 [Preprint]. 2018 [cited 2019 May 30]. Available from: https://arxiv.org/abs/1803.02432.

[17] Wold H. Estimation of Principal Components and Related Models by Iterative Least squares In: Multivariate Analysis. New York: Academic Press; 1966 p. 391–420.

[18] Fisher RA. The Use of Multiple Measurements in Taxonomic Problems. Annals of Eugenics. 1936;7(2):179–188. 10.1111/j.1469-1809.1936.tb02137.x

[19] Goldberger J, Roweis S, Hinton G, Salakhutdinov R. Neighbourhood Components Analysis. In: Proceedings of the 17th International Conference on Neural Information Processing Systems. Cambridge, MA: MIT Press; 2004. p. 513–520.

[20] Parviainen E. Deep Bottleneck Classifiers in Supervised Dimension Reduction. In: Proceedings of the 20th International Conference on Artificial Neural Networks: Part III. ICANN'10. Berlin, Heidelberg: Springer-Verlag; 2010. p. 1–10.

[21] Hurley JR, Cattell RB. The procrustes program: Producing direct rotation to test a hypothesized factor structure. Behavioral Science. 1962;7(2):258–262. 10.1002/bs.3830070216

[22] Escoufier Y. L'analyse conjointe de plusieurs matrices de données. Biométrie et temps. 1980 p. 59–76.

[23] Lavit C, Escoufier Y, Sabatier R, Traissac P. The ACT (STATIS method). Computational Statistics & Data Analysis. 1994;18(1):97–119. 10.1016/0167-9473(94)90134-1

[24] Abdi H, O'Toole AJ, Valentin D, Edelman B. DISTATIS: The Analysis of Multiple Distance Matrices. 2005 IEEE Computer Society Conference on Computer Vision and Pattern Recognition (CVPR'05)—Workshops; 2005. San Diego, CA. IEEE. p. 42–42.

[25] Kassambara A, Mundt F. factoextra: Extract and Visualize the Results of Multivariate Data Analyses. Version 1.0.5 [software]. 2017 [cited 2019 May 30]. Available from: https://CRAN.R-project.org/package=factoextra.

[26] Love MI, Huber W, Anders S. Moderated estimation of fold change and dispersion for RNA-seq data with DESeq2. Genome Biology. 2014;15(12):550 10.1186/s13059-014-0550-8 25516281 PMC4302049

[27] Robinson MD, McCarthy DJ, Smyth GK. edgeR: a Bioconductor package for differential expression analysis of digital gene expression data. Bioinformatics. 2010;26(1):139–140. 10.1093/bioinformatics/btp616 19910308 PMC2796818

[28] Laubscher NF. On Stabilizing the Binomial and Negative Binomial Variances. Journal of the American Statistical Association. 1961;56(293):143–150. 10.1080/01621459.1961.10482100

[29] Burbidge JB, Magee L, Robb AL. Alternative Transformations to Handle Extreme Values of the Dependent Variable. Journal of the American Statistical Association. 1988;83(401):123–127. 10.1080/01621459.1988.10478575

[30] Huber W, von Heydebreck A, Sültmann H, Poustka A, Vingron M. Variance stabilization applied to microarray data calibration and to the quantification of differential expression. Bioinformatics. 2002;18(Suppl 1):S96–S104. 12169536 10.1093/bioinformatics/18.suppl_1.s96

[31] Escofier B, Pagès J. Multiple factor analysis (AFMULT package). Computational Statistics & Data Analysis. 1994;18(1):121–140. 10.1016/0167-9473(94)90135-X

[32] Guttman L. The quantification of a class of attributes: A theory and method of scale construction. The Prediction of Personal Adjustment. 1941; p. 319–348.

[33] Gifi A. Nonlinear multivariate analysis. Chichester, New York: Wiley; 1990.

[34] Meulman JJ, Heiser WJ, et al SPSS Categories 10.0. Chicago: SPSS Incorporated; 1999.

[35] Linting M, Meulman JJ, Groenen PJF, van der Koojj AJ. Nonlinear principal components analysis: Introduction and application. Psychological Methods. 2007;12(3):336–358. 10.1037/1082-989X.12.3.336 17784798

[36] Borg I, Groenen PJF. Modern Multidimensional Scaling: Theory and Applications. New York, NY: Springer; 2005.

[37] Kleindessner M, Luxburg U. Uniqueness of Ordinal Embedding. In: Balcan MF, Feldman V, Szepesvári C, editors. Proceedings of The 27th Conference on Learning Theory. vol. 35 of Proceedings of Machine Learning Research. Barcelona, Spain: PMLR; 2014. p. 40–67.

[38] Kleindessner M, von Luxburg U. Lens Depth Function and k-Relative Neighborhood Graph: Versatile Tools for Ordinal Data Analysis. Journal of Machine Learning Research. 2017;18(58):1–52.

[39] Mikolov T, Chen K, Corrado G, Dean J. Efficient Estimation of Word Representations in Vector Space. arXiv:1301.3781 [Preprint]. 2013 [cited 2019 May 30]. Available from: https://arxiv.org/abs/1301.3781.

[40] Du J, Jia P, Dai Y, Tao C, Zhao Z, Zhi D. Gene2Vec: Distributed Representation of Genes Based on Co-Expression. BioRxiv [Preprint]. 2018 [cited 2019 May 30]. Available from: https://www.biorxiv.org/content/10.1101/286096v2. 10.1186/s12864-018-5370-x PMC6360648 30712510

[41] Gabriel KR. The Biplot Graphic Display of Matrices with Application to Principal Component Analysis. Biometrika. 1971;58(3):453–467.

[42] Jolicoeur P, Mosimann JE. Size and shape variation in the painted turtle. A principal component analysis. Growth. 1960;24:339–54. 13790416

[43] Husson F, Josse J, Pagès J. Principal component methods-hierarchical clustering-partitional clustering: why would we need to choose for visualizing data? Rennes, France: Agrocampus Ouest; 2010.

[44] Diaconis P, Goel S, Holmes S. Horseshoes in Multidimensional Scaling and Local Kernel Methods. The Annals of Applied Statistics. 2008;2(3):777–807.

[45] Trench WF. Spectral distribution of generalized Kac–Murdock–Szego matrices. Linear Algebra and its Applications. 2002;347(1):251–273. 10.1016/S0024-3795(01)00561-4

[46] Reid JE, Wernisch L. Pseudotime estimation: deconfounding single cell time series. Bioinformatics. 2016;32(19):2973–2980. 10.1093/bioinformatics/btw372 27318198 PMC5039927

[47] Campbell KR, Yau C. Uncovering pseudotemporal trajectories with covariates from single cell and bulk expression data. Nature Communications. 2018;9(1):2442 10.1038/s41467-018-04696-6 29934517 PMC6015076

[48] Campbell K, Yau C. Probabilistic modeling of bifurcations in single-cell gene expression data using a Bayesian mixture of factor analyzers. Wellcome Open Research. 2017;2(19). 10.12688/wellcomeopenres.11087.1 28503665 PMC5428745

[49] Nguyen LH, Holmes S. Bayesian Unidimensional Scaling for visualizing uncertainty in high dimensional datasets with latent ordering of observations. BMC Bioinformatics. 2017;18(10):394 10.1186/s12859-017-1790-x 28929970 PMC5606221

[50] Forina M, Leardi R, C A, Lanteri S. PARVUS: An Extendable Package of Programs for Data Exploration. Amsterdam, the Netherlands: Elsevier; 1988.

[51] Dheeru D, Karra Taniskidou E. UCI Machine Learning Repository [Internet]. Irvine, CA: University of California, School of Information and Computer Science; 2017 [cited 2019 May 30]. Available from: http://archive.ics.uci.edu/ml.

[52] Ray B, Henaff M, Ma S, Efstathiadis E, Peskin E, Picone M, et al Information content and analysis methods for multi-modal high-throughput biomedical data. Scientific Reports. 2014;4 10.1038/srep04411 24651673 PMC3961740

[53] Abdi H, Williams LJ, Valentin D, Bennani-Dosse M. STATIS and DISTATIS: optimum multitable principal component analysis and three way metric multidimensional scaling. Wiley Interdisciplinary Reviews: Computational Statistics. 2012;4(2):124–167. 10.1002/wics.198

[54] L'Hermier des Plantes H. Structuration des tableaux à trois indices de la statistique: théorie et application d'une méthode d'analyse conjointe. Montpellier, France: Université des sciences et techniques du Languedoc; 1976.

[55] Meng C, Zeleznik OA, Thallinger GG, Kuster B, Gholami AM, Culhane AC. Dimension reduction techniques for the integrative analysis of multi-omics data. Brief Bioinform. 2016;17(4):628–641. 10.1093/bib/bbv108 26969681 PMC4945831

[56] Wattenberg M, Viégas F, Johnson I. How to Use t-SNE Effectively. Distill. 2016 10.23915/distill.00002

[57] Cao Y, Wang L. Automatic Selection of t-SNE Perplexity. arXiv:1708.03229 [Preprint]. 2017 [cited 2019 May 30]. Available from: https://arxiv.org/abs/1708.03229.

[58] Debruyne M, Hubert M, Horebeek JV. Detecting influential observations in Kernel PCA. Computational Statistics & Data Analysis. 2010;54(12):3007–3019. 10.1016/j.csda.2009.08.018

[59] Sandve GK, Nekrutenko A, Taylor J, Hovig E. Ten Simple Rules for Reproducible Computational Research. PLoS Comput Biol. 2013;9(10):1–4. 10.1371/journal.pcbi.1003285 24204232 PMC3812051

