# Supplementary material for: Ten quick tips for effective dimensionality reduction
Source: PLoS Comput Biol. 2019 Jun 20;15(6):e1006907. doi: 10.1371/journal.pcbi.1006907 (PMC6586259; doi:10.1371/journal.pcbi.1006907)
Supplement: S1 Text — (PDF) [file pcbi.1006907.s002.pdf]

Ten quick tips for dimensionality reduction: figures for the paper

Lan Huong Nguyen

1 April 2019

Contents

1 Scree plot . . . 2
2 Aspect Ratio . . . 4
3 External Information . . . 9
4 Latent structure . . . 16
5 Distatis . . . 18
6 Procrustes . . . 20
7 Eigenvalues instability . . . 24
8 Striking image . . . 26

In this document we provide code to generate all figures included in the paper. Most of plots are generated using the `factoextra` package by Kassambara and Mundt.

```
library(factoextra)
library(lattice)
library(tidyverse)
library(gridExtra)
library(viridis)
theme_set(theme_minimal())
theme_update(
  text = element_text(size = 20)
)
```

1 Scree plot

Below we generate 5-dimensional data and projected onto 500-dimensional space using multiplication by an orthogonal matrix.

```
set.seed(4756)
n <- 1000
d <- 5
p <- 500
sigma <- 1
R1 <- matrix(rnorm(n*d), nrow = n)
R2 <- matrix(rnorm(p*d), nrow = p)
U <- qr.Q(qr(R1))
V <- qr.Q(qr(R2))
sv <- sqrt(n)*c(1.8, 2.1, 2.5, 3.3, 4)
X <- U %*% diag(sv) %*% t(V)
Y <- X + matrix(rnorm(n*p, sd = sigma), ncol = p)

# Compute PCA
pca.res <- prcomp(Y, scale. = FALSE)
```

A scree plot shows percentage of variance explained by consecutive principal components (Fig.1). Percentage of variance explained is equal to the normalized eigenvalues of the covariance matrix used in PCA.

```
var_explained <- 100*(pca.res$sdev^2)/sum(pca.res$sdev^2)
var_explained[1:10]
##  [1] 3.2404152 2.3129465 1.4039711 1.1291411 0.8330401 0.5300693 0.5212096 0.5131639 0.5096728 0.5046514
```

```
fviz_eig(pca.res) + ggtitle("") +
  theme(text = element_text(size = 20))
```

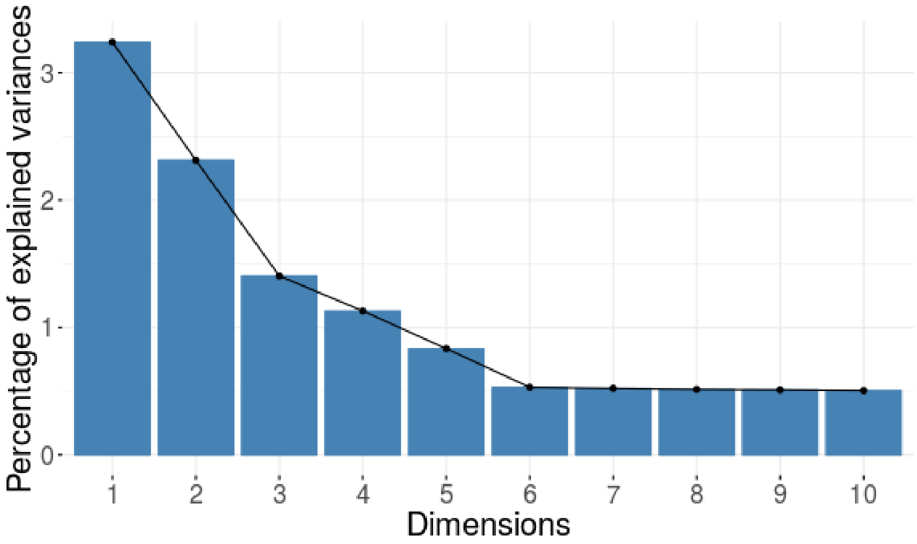

You can also inspect the histogram of the eigenvalues and the fitted Marchenko-Pastur distribution. The eigenvalues to the right of the fitted curve correspond to the signal, i.e. are larger than the eigenvalues expected if data (with the same dimensionality) was pure noise.

```
library(RMTstat)
df <- data.frame(sv = pca.res$sdev, eig = pca.res$sdev^2)
mp <- data.frame(eig = seq(0, max(df$eig), length.out = 100)) %>%
  mutate(mp = dmp(eig, svr = n/p, var = sigma^2))
ggplot(df, aes(x = eig)) +
  geom_histogram(
    bins = 80, fill = "grey30", color = "white", lwd =  0.2,
    aes(y = ..density..)) +
  geom_line(data = mp, aes(y = mp-0.01), color = "royalblue", lwd = 1)
```

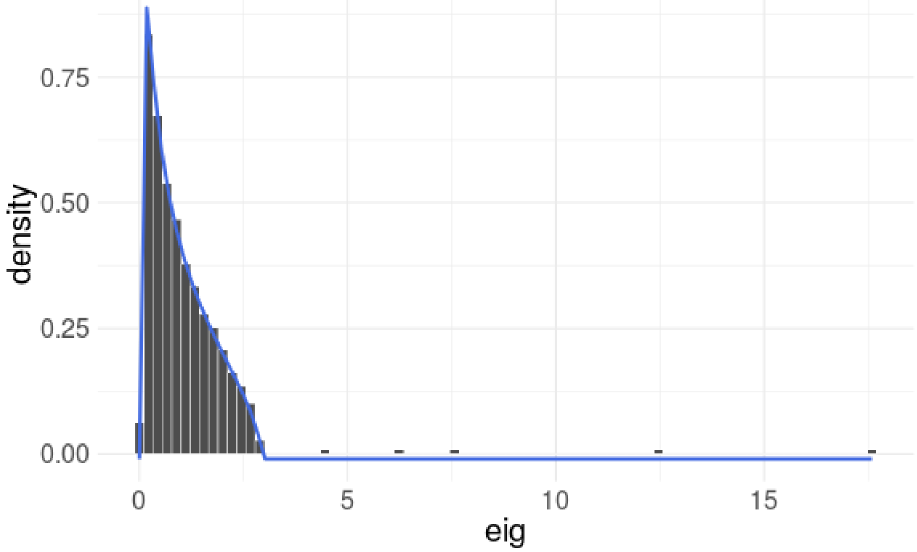

2 Aspect Ratio

We generate data from 2 Gaussian 2D clusters. We delete some observations around x-axis (pretend they are missing). Then, we project the observations to 50 dimensions (pretend we observed high-dimensional data).

```
set.seed(834759)
N <- 500
X <- cbind(
  x1 = c(rnorm(N, mean = 0, sd = 4.5),
         rnorm(N, mean = 20, sd = 4.5)),
  y = c(rnorm(N, mean = 0, sd = 1),
        rnorm(N, mean = 0, sd = 1))
)

class <-  rep(c("A", "B"), each  = N)

thresh <- 0.2
class <- class[abs(X[, 2]) > thresh]
X <- X[abs(X[, 2]) > thresh, ]

n <- nrow(X)
p <- 50
tmp <- matrix(rnorm(n*n, mean = 0, sd = 1), ncol = n)
tmp.qr <- qr(tmp)
Q <- qr.Q(tmp.qr, complete=TRUE)
Q <- Q[, 1:p]

Y <- X %*% Q[1:2, ]
pca.res <- prcomp(Y, scale = TRUE)
```

The original 2 dimensional clusters have different variances along the X1 and X2 axis (more variability along the first axis). Some data are missing around x-axis, but the red and blue clusters are still relatively well separated.

```
df <- data.frame(X, class)
colnames(df) <- c("x", "y","label")
ggplot(df, aes(x = x, y = y, color = label)) +
  geom_point(size = 1) + coord_fixed() +
  scale_color_brewer(name = "Group", palette = "Set1") +
  theme_minimal()
```

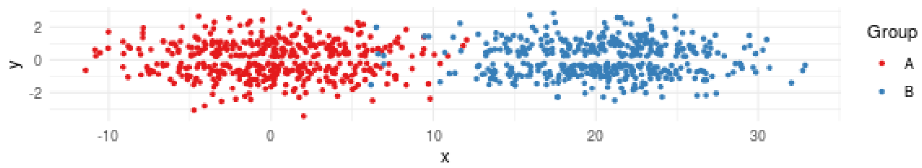

Plots below are included in Fig. 2.

We compute the observed 50D data to the first 2 PCs, but here we use an arbitrary aspect ratio producing an (incorrect) rectangular plot.

```r
fviz_pca_ind(pca.res, geom = "point")  +
  ggtitle("") + coord_fixed(4) +
  theme(text = element_text(size = 30))
```

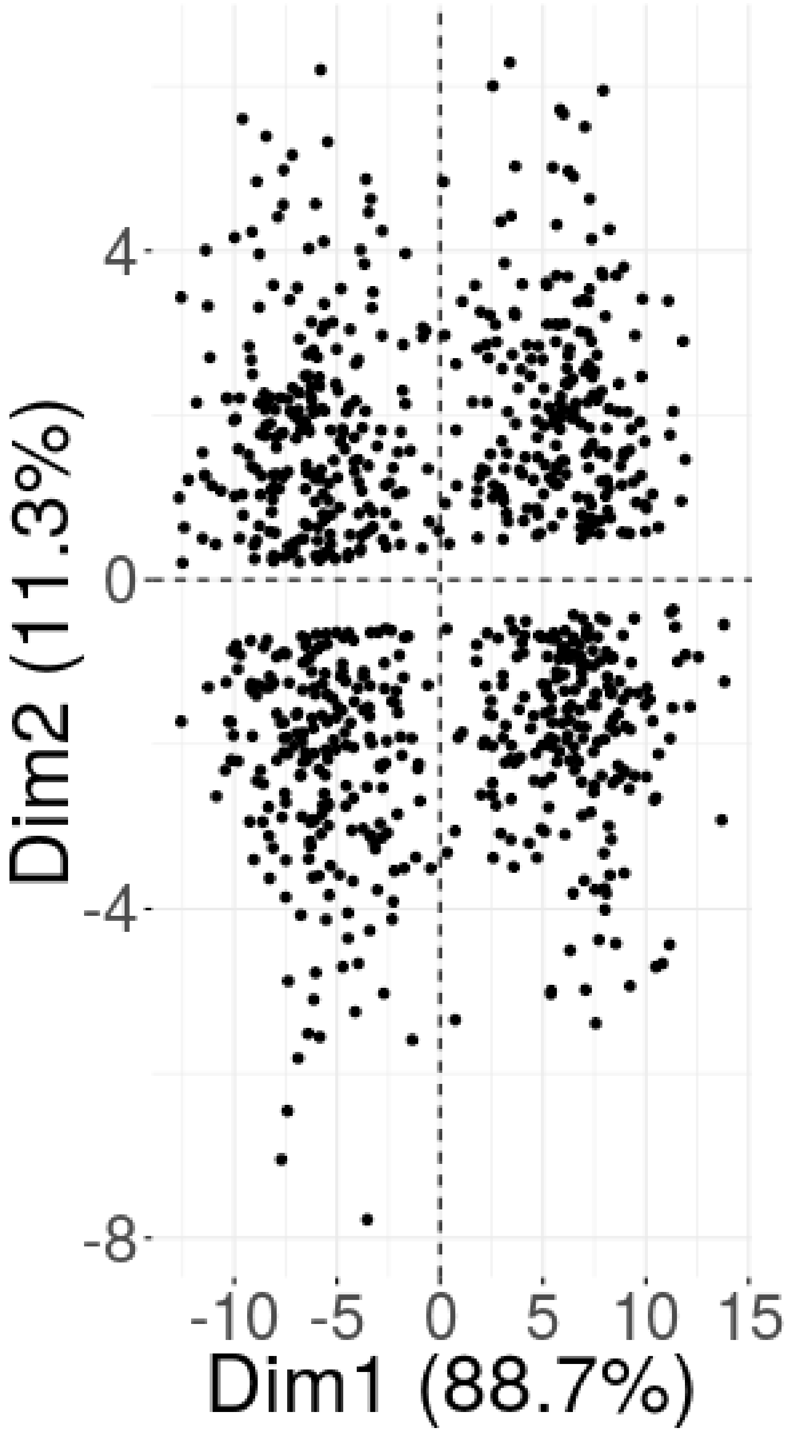

```
fviz_pca_ind(pca.res, geom = "point", col.ind = df$label,
             pointsize = 2.5, invisible="quali") + coord_fixed(4) +
  scale_shape_manual(name = "Group", values = c(16, 16)) +
  scale_color_brewer(name = "Group", palette = "Set1") +
  ggtitle("") +
  theme(text = element_text(size = 30))
```

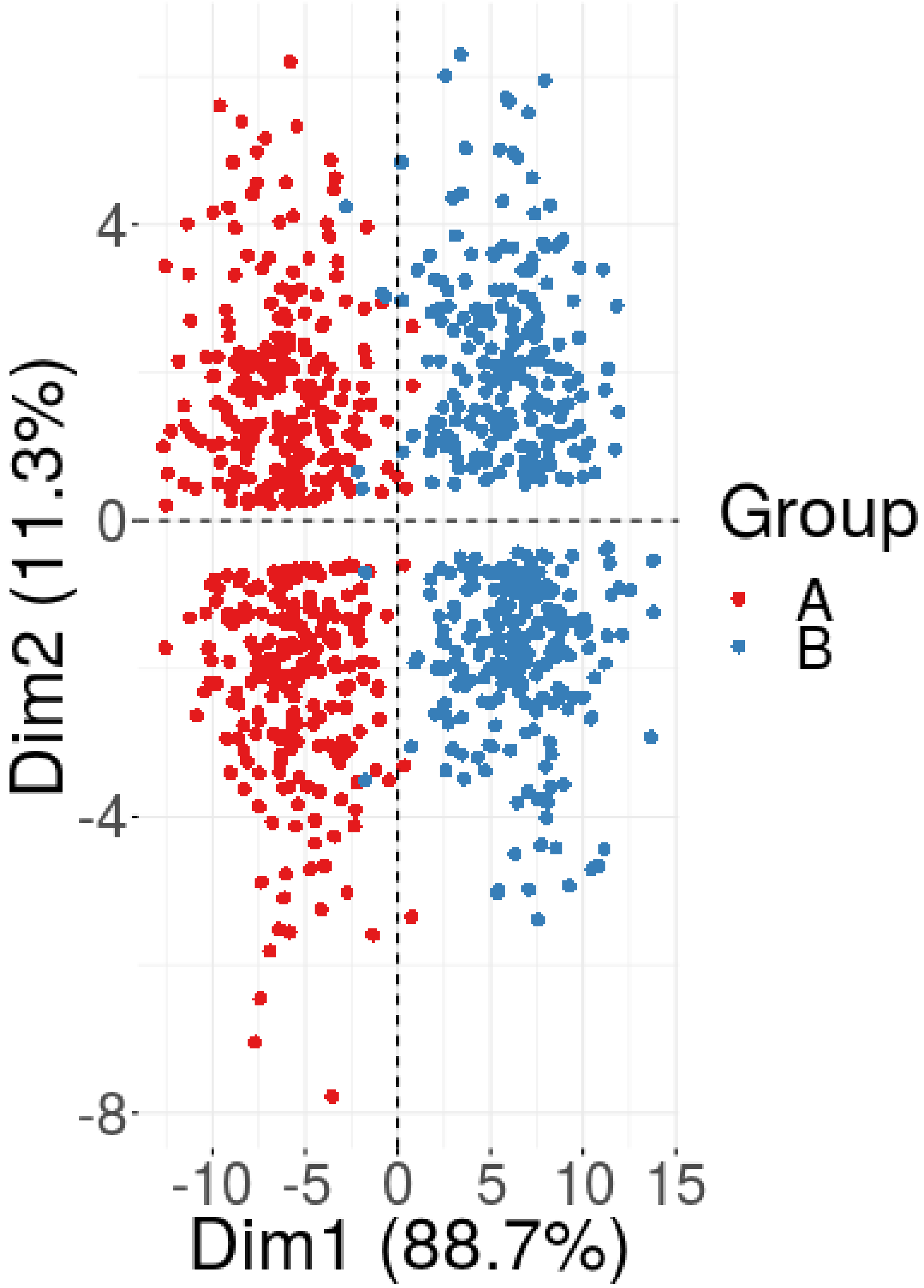

Similarly, one might produce a strictly square plot (set code chunk settings to equal height and with: `fig.width=8, fig.height=8`). The following plot has still an incorrect aspect ratio.

```r
fviz_pca_ind(pca.res, geom = "point")  + xlim(NA, 15) +
  ggtitle("") + theme(text = element_text(size = 30))
```

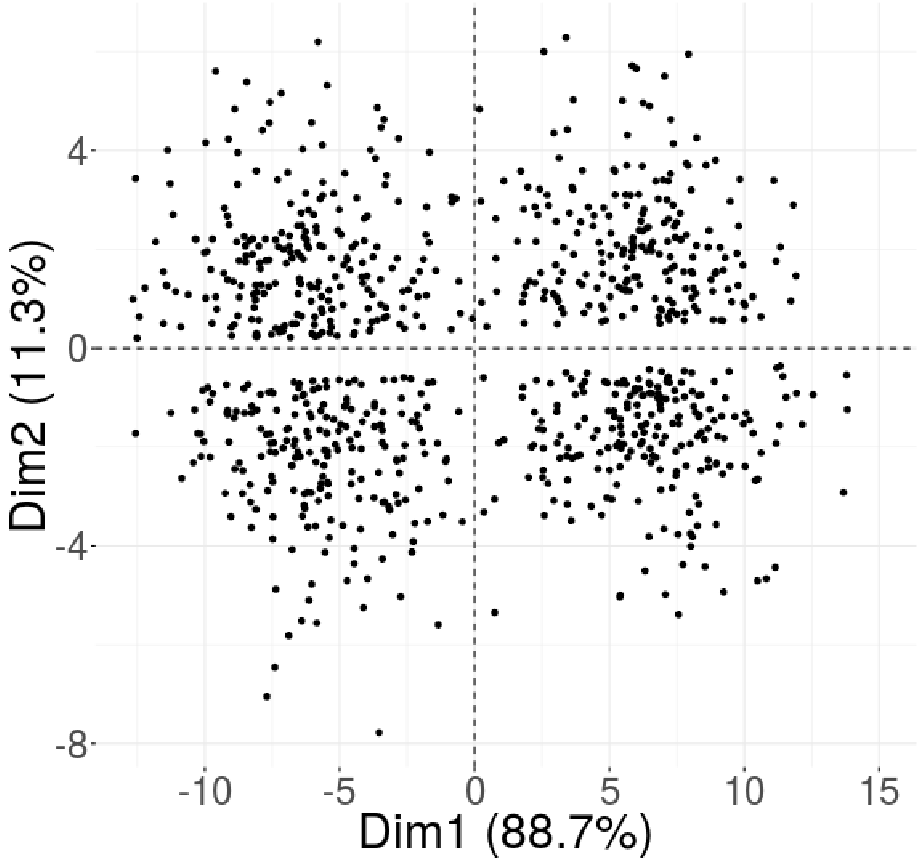


```r
fviz_pca_ind(pca.res, geom = "point", col.ind = df$label,
             pointsize = 2.5, invisible="quali") +
  scale_shape_manual(name = "Group", values = c(16, 16)) +
  scale_color_brewer(name = "Group", palette = "Set1") +
  ggtitle("") + xlim(NA, 15) +
  theme(text = element_text(size = 30))
```

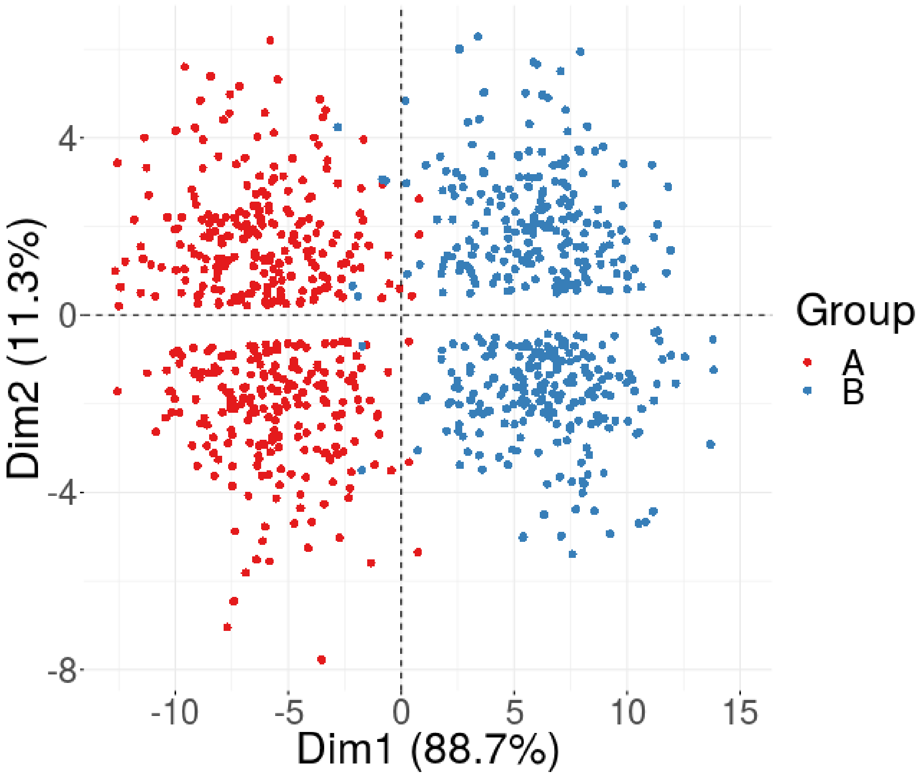

A correct ratio, is the one that reflects the ration between the variance explained by each axis. This can be done by adding `+ coord_fixed(1)` to a ggplot object.

```
fviz_pca_ind(pca.res, geom = "point") + coord_fixed() +
  ggtitle("") + xlim(NA, 15) +
  theme(text = element_text(size = 30))
```

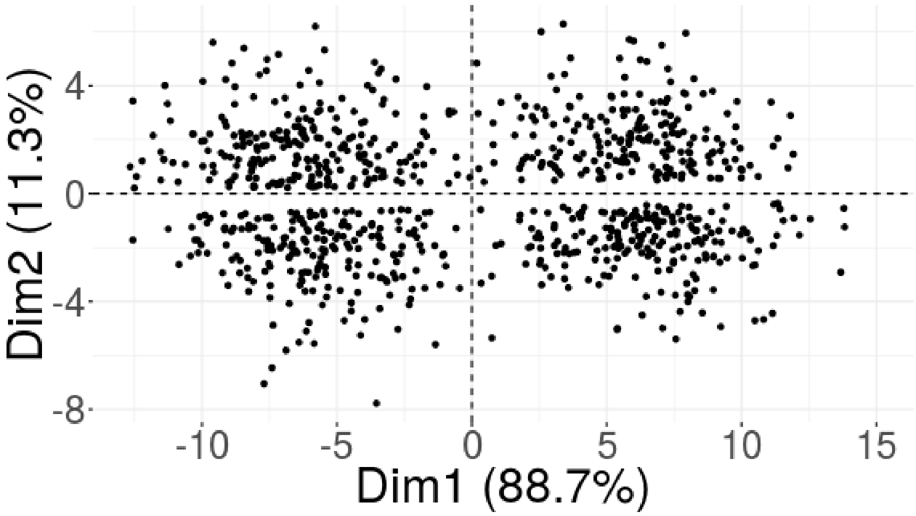


```
fviz_pca_ind(pca.res, geom = "point", col.ind = df$label, pointsize = 2.5,
             invisible="quali") +
  scale_shape_manual(name = "Group", values = c(16, 16)) +
```

```
    scale_color_brewer(name = "Group", palette = "Set1") +
    coord_fixed() +  ggtitle("") +
    theme(text = element_text(size = 30))
```

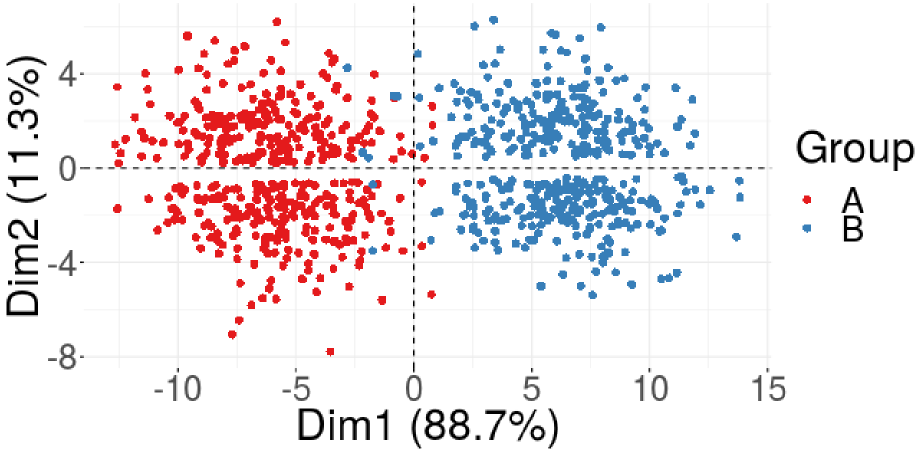

3 External Information

Here, we use the wine dataset as example. The variables include the chemical properties and composition of the wines. Wine class labels for grape varieties (59 Barolo, 71 Grignolino, 48 Barbera) are also available.

```
url <- "https://archive.ics.uci.edu/ml/machine-learning-databases/wine/wine.data"
wine <- read_csv(url, col_names = FALSE)
colnames(wine) <- c("class", "Alcohol", "MalicAcid", "Ash", "AlcAsh", "Mg",
                    "Phenols", "Flav", "NonFlavPhenols", "Proa", "Color",
                    "Hue", "OD", "Proline")
head(wine)
## # A tibble: 6 x 14
##   class Alcohol MalicAcid   Ash AlcAsh    Mg Phenols  Flav NonFlavPhenols  Proa Color   Hue    OD Proline
##   <dbl>   <dbl>     <dbl> <dbl>  <dbl> <dbl>   <dbl> <dbl>          <dbl> <dbl> <dbl> <dbl> <dbl>   <dbl>
## 1     1    14.2      1.71  2.43   15.6   127    2.8   3.06           0.28  2.29  5.64  1.04  3.92    1065
## 2     1    13.2      1.78  2.14   11.2   100    2.65  2.76           0.26  1.28  4.38  1.05  3.4     1050
## 3     1    13.2      2.36  2.67   18.6   101    2.8   3.24           0.3   2.81  5.68  1.03  3.17    1185
## 4     1    14.4      1.95  2.5    16.8   113    3.85  3.49           0.24  2.18  7.8   0.86  3.45    1480
## 5     1    13.2      2.59  2.87   21     118    2.8   2.69           0.39  1.82  4.32  1.04  2.93     735
## 6     1    14.2      1.76  2.45   15.2   112    3.27  3.39           0.34  1.97  6.75  1.05  2.85    1450
# OD variable is = OD280/OD315 of diluted wines
```

```
# note that Nebbiolo is a grape variety for Barolo
wine.class <- factor(
    wine$class,
    levels = c(1, 2, 3),
    labels = c("Nebbiolo", "Grignolino", "Barbera"))
wine <- wine[,-1]
table(wine.class)
```

```
## wine.class
##   Nebbiolo Grignolino    Barbera
##         59         71         48
```

Scree plot for wine data:

```
winePCA <- prcomp(wine, scale = TRUE)
fviz_eig(winePCA)
```

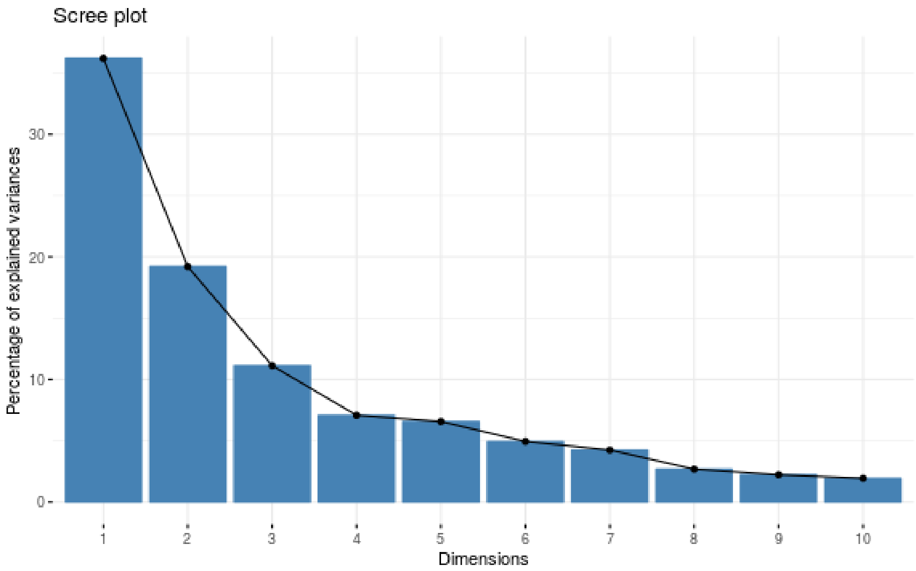

Correlation circle, feature projection plot, for Fig. 3 (a):

```
fviz_pca_var(winePCA, labelsize = 7) +
  ggtitle("") + theme(text = element_text(size = 25))
```

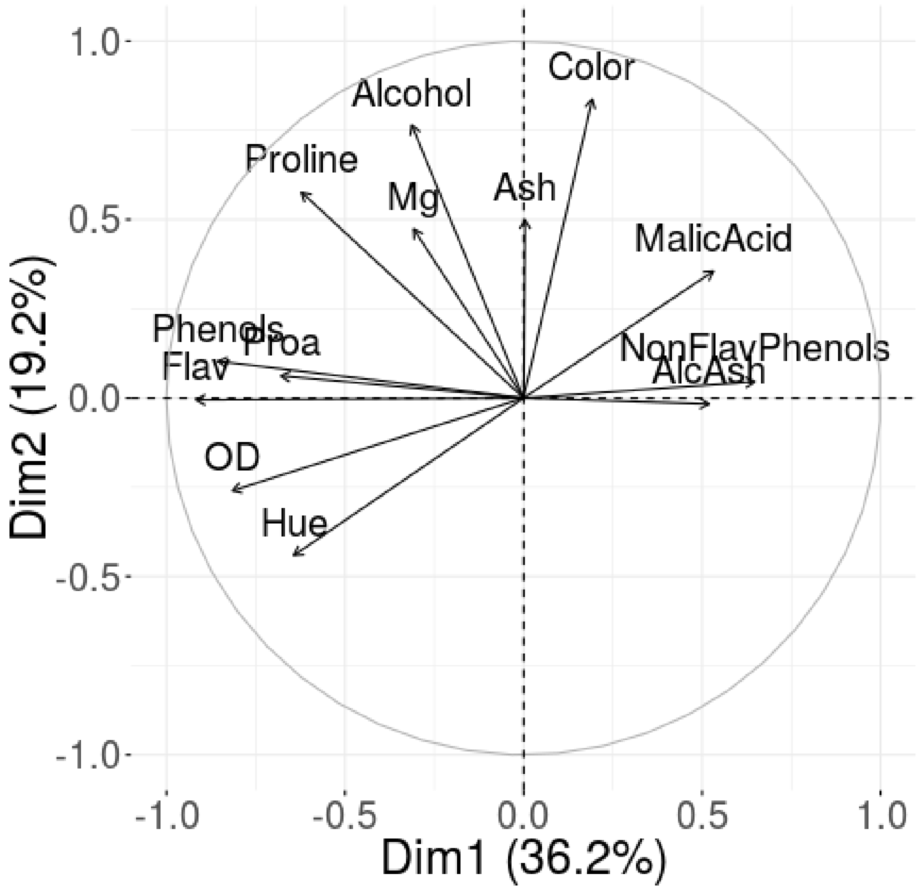

Contribution plot for Fig. 3(b)

```
fviz_contrib(winePCA, choice = "var", axes = 1) +
  ggtitle("") + theme(text = element_text(size = 25))
```

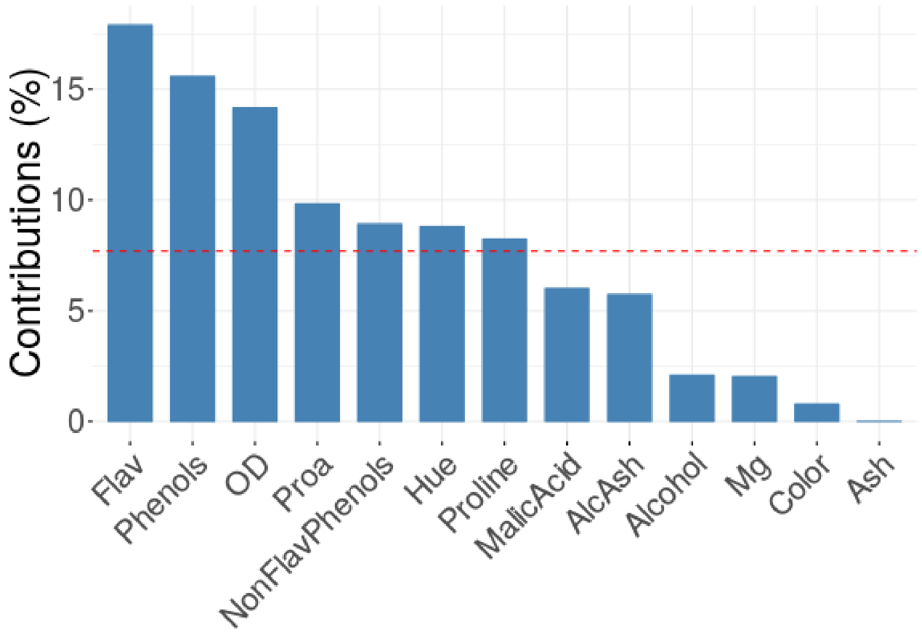

Sample projection plot:

```
#eig_ratio <- winePCA$eig[2]/winePCA$eig[1]
fviz_pca_ind(winePCA) +
  coord_fixed() + theme(text = element_text(size = 25))
```

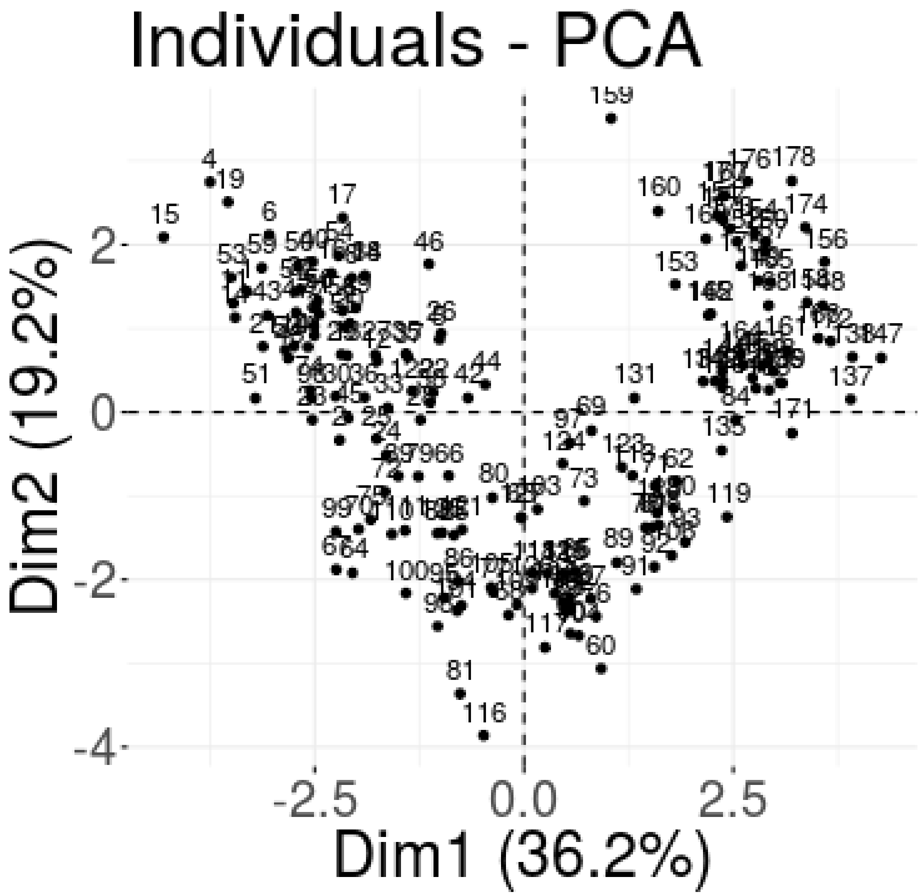

Biplot, Fig. 4.

```
fviz_pca_biplot(pointsize = 2.5,
  winePCA, geom = "point",
  labelsize = 8, col.var = "#c07d44") +
  coord_fixed() + ggtitle("") +
  ylim(-4.5, 4.5) + xlim(-5, 5) +
  theme(text = element_text(size = 25))
```

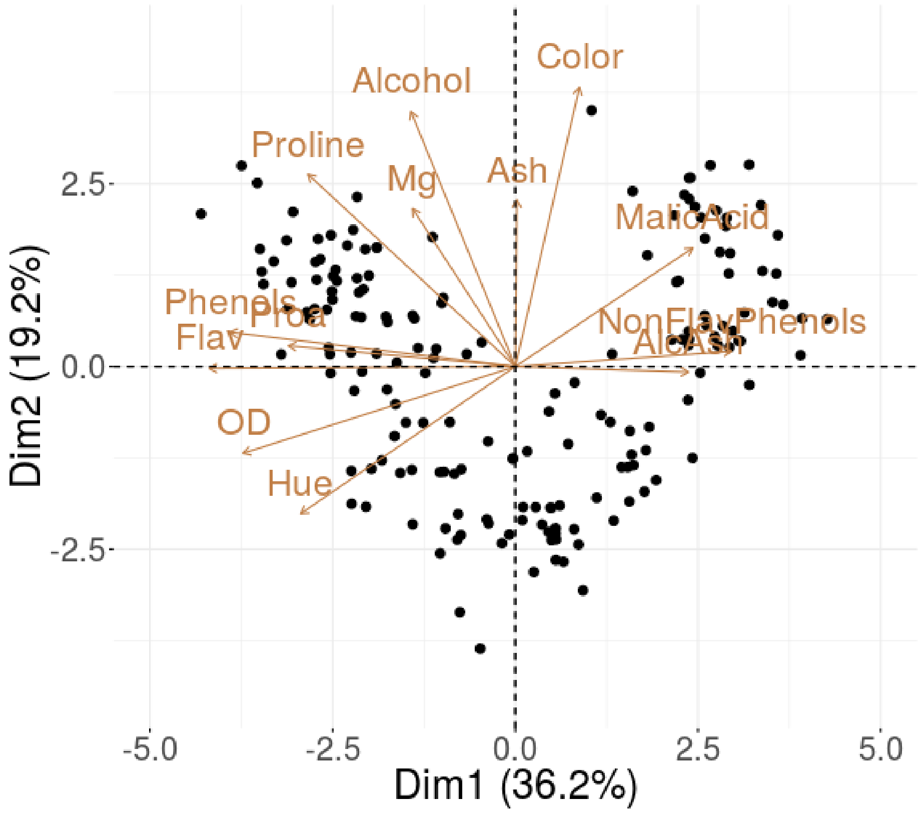

Sample plot with class memberships, Fig. 6(a):

```
fviz_pca_ind(winePCA, col.ind = wine.class, pointsize = 2.5,
             ellipse.type = "convex", addEllipses = TRUE, labelsize = 6) +
    scale_fill_brewer(name = "Grape variety", palette = "Set1") +
    scale_color_brewer(name = "Grape variety", palette = "Set1") +
    scale_shape_manual(name = "Grape variety", values = c(16, 16, 16)) +
    coord_fixed() +  ggtitle("") +  ylim(-4, 4) +
    guides(color = "none", fill = "none", shape = "none") +
    theme(text = element_text(size = 25))
```

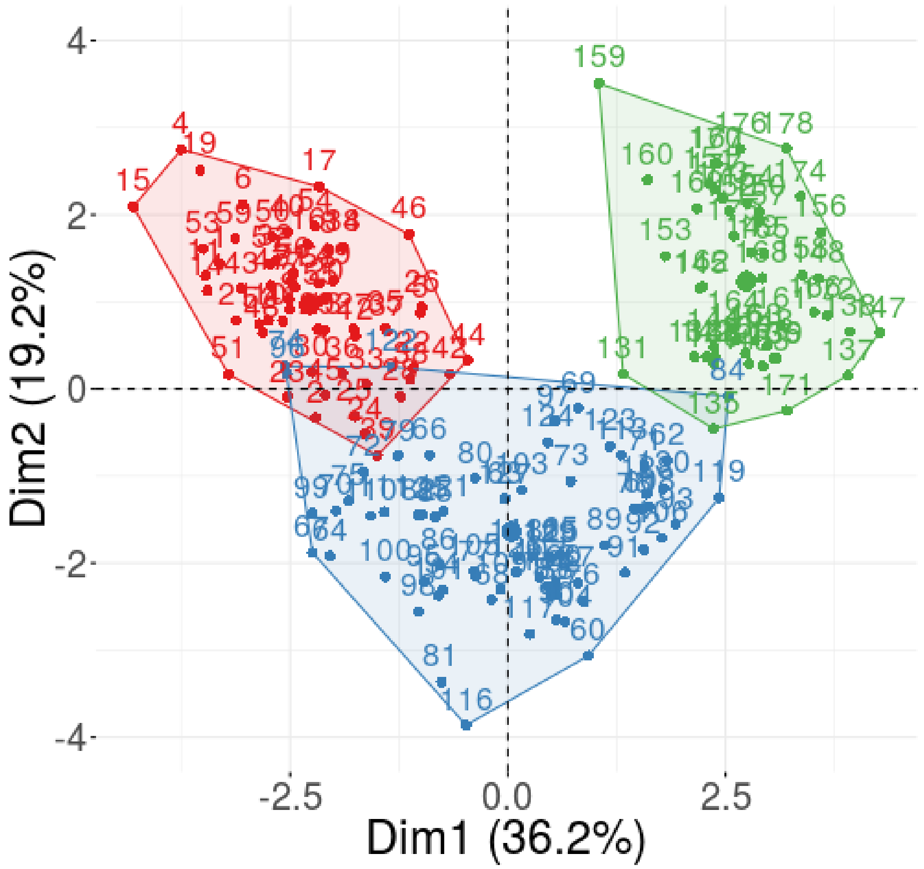

Biplot with sample group ellipsoids, Fig. 6(b)

```
fviz_pca_biplot(
  winePCA, geom = "point",
  col.ind = wine.class,
  col.var = "#c07d44", pointsize = 2.5, labelsize = 7,
  addEllipses = TRUE, ellipse.level = 0.7) +
  coord_fixed() + ggtitle("") + ylim(-4, 4) +
  scale_fill_brewer(name = "Grape variety", palette = "Set1") +
  scale_color_brewer(name = "Grape variety", palette = "Set1") +
  scale_shape_manual(name = "Grape variety", values = c(16, 16, 16)) +
  theme(text = element_text(size = 25))
```

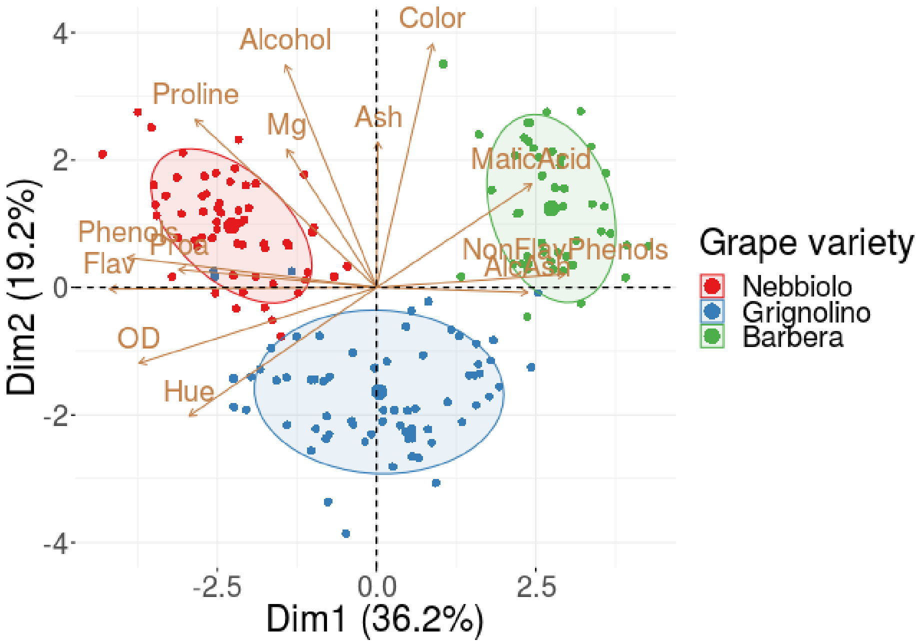

4 Latent structure

We generate five clusters of data points in 2D, and then project them to 10D.

```
set.seed(98574) #7465
m <- 100
d <- 2
p <- 10
k <- 5
sigma <- 1
muMax <- 25
mu <- matrix(runif(k*d, max = muMax), ncol = d)

X <- lapply(seq_len(k), function(i) {
  ix <- rep(1, m) %*% t(mu[i, ]) + matrix(sigma*rnorm(m*d), ncol = d)
  return(ix)
})
names(X) <- paste0("Cluster", 1:k)
X.df <- plyr::ldply(X, function(x) x, .id = "Cluster")
X <- as.matrix(X.df[, -1])
#Q <- qr.Q(qr( matrix(rnorm(d*p), nrow = p)))
Y <- X  %*% matrix(rnorm(d*p), ncol = p)
```

```
pca.res <- prcomp(Y)
fviz_eig(pca.res)
```

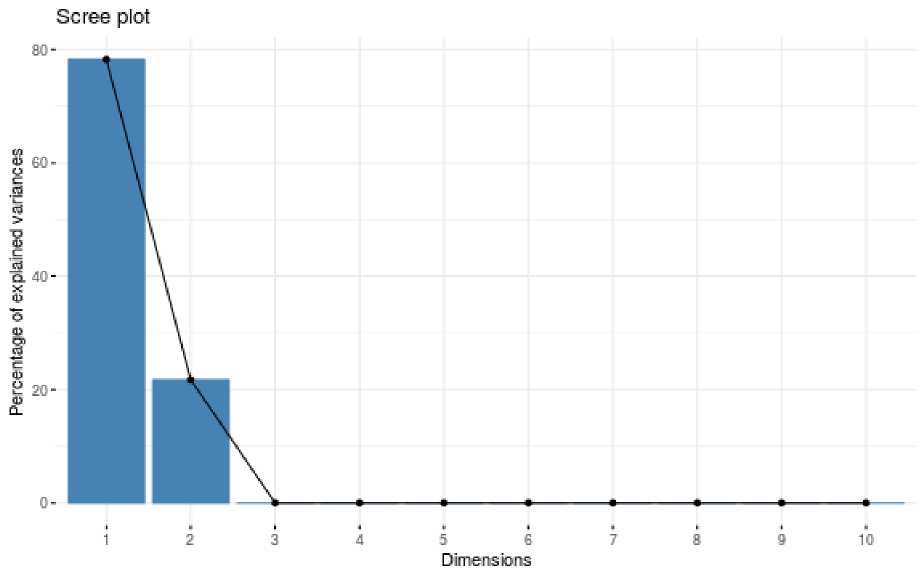

The PCA plot shows clusters in Fig. 5(a).

```
fviz_pca_ind(pointsize = 2.5,
    pca.res, col.ind = X.df$Cluster, geom = "point") +
    guides(color=guide_legend(title="Membership"),
           shape=guide_legend(title="Membership")) +
    coord_fixed() +
  ggtitle("") + theme(text = element_text(size = 25))
```

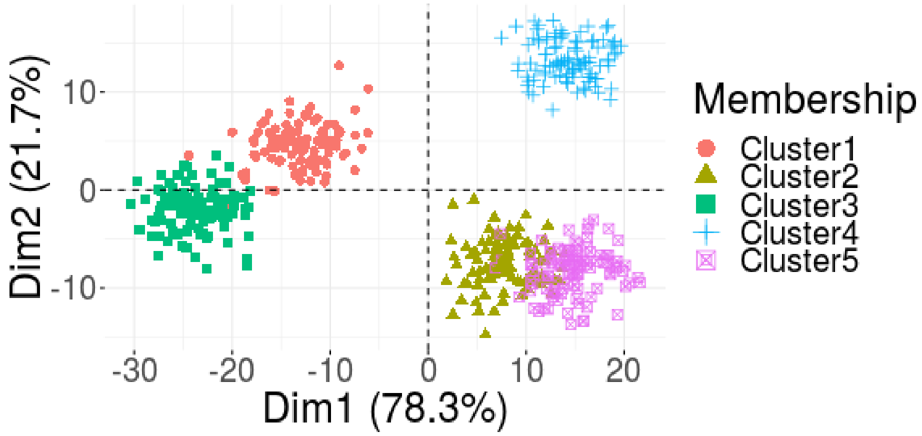

Then, we simulate data generated from a smooth continuous gradient (modeled as a Gaussian Process Latent Variable Model).

```
set.seed(7365)
n <- 500
p <- 10
sigma <- 0.01
t <- sort(c(seq(0, 1, length.out = 100), runif(n-100)))
K <- exp(-as.matrix(dist(t))) + diag(rep(sigma, n))
Q <- qr.Q(qr(matrix(rnorm(n*n), ncol = n)))
E <- 0.05*matrix(rnorm(n*n), ncol = n)
```

```
X <- (K + E) %*% Q[, seq_len(p)]
```

The scree plot:

```
pca.res <- prcomp(X)
fviz_eig(pca.res)
```

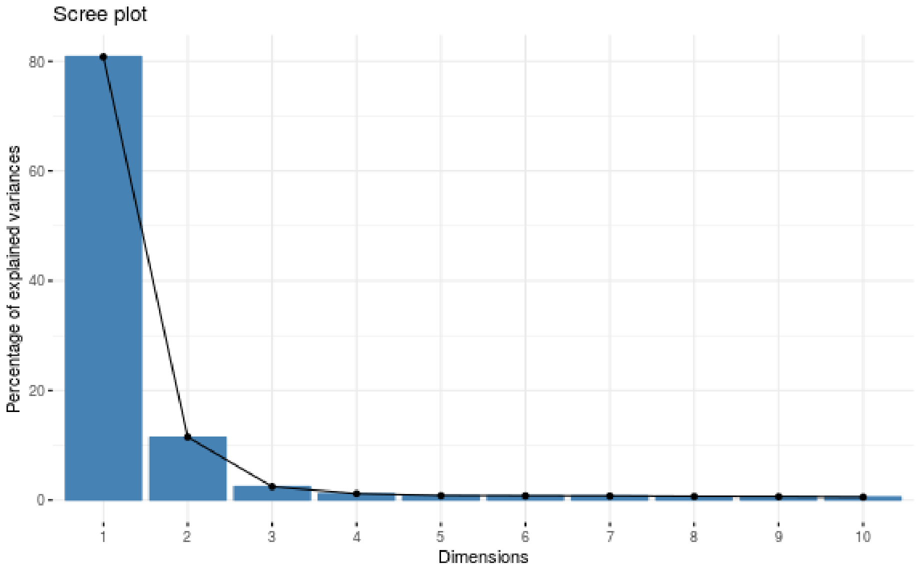

Continuous gradients shows up as a horseshoe on a PCA plot, Fig. 5(b)

```
fviz_pca_ind(pca.res, col.ind = t, geom = "point", pointsize = 2.5) +
  coord_fixed() +
  scale_color_viridis(name = "Depth") +
  ggtitle("") + theme(text = element_text(size = 25))
```

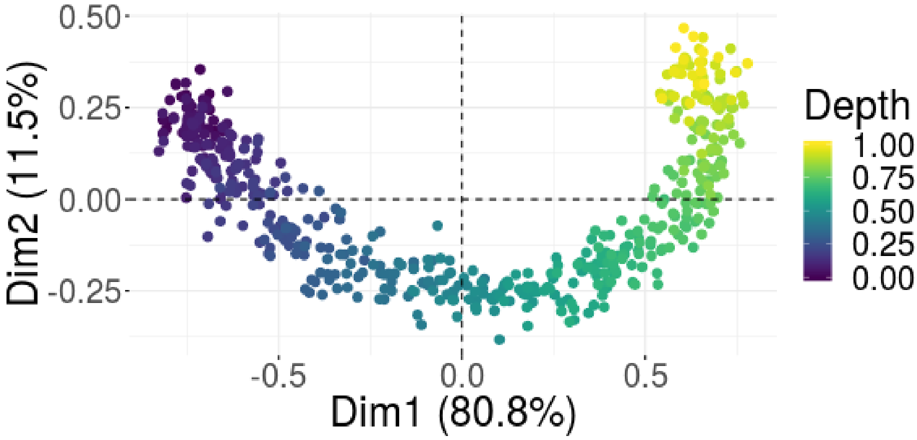

5 Distatis

We generate synthetic data “as if coming from 5 separate data tables”.

```r
set.seed(123)
library(ade4)
n_copies <- 5
n <- 20
p <- 5
sigma <- 5
M <- matrix(rnorm(n*p, sd = sigma), nrow = n)
E.lst <- lapply(seq_len(n_copies), function(x) matrix(rnorm(n*p), nrow = n))
M.lst <- lapply(E.lst, function(E) {data.frame(M + E)})
# ktab <- ktab.list.df(M.lst)
# statis.res <- statis(ktab, scannf = FALSE, nf = 2)
```

Then, we compute 5 distance matrices:

```r
D.lst <- lapply(M.lst, function(x) as.matrix(dist(x)))
D.arr <- abind::abind(D.lst, along = 3)
distatis.res <- DistatisR::distatis(D.arr, nfact2keep = n)
df <- distatis.res$res4Splus$PartialF %>%
  reshape2::melt(varnames = c("SampleID", "FactorID", "TableID")) %>%
  spread(key = "FactorID", value = "value") %>%
  mutate(SampleID = factor(SampleID))
consensus.df <- data.frame(
  SampleID = factor(seq_len(nrow(distatis.res$res4Splus$F))),
  distatis.res$res4Splus$F, check.names = FALSE)
```

Fig. 7 shows DISTATIS results similar to what can be obtained for multi-table data.

```r
cols <- colorRampPalette(RColorBrewer::brewer.pal(9, "Set1"))(n)
ggplot(
  data = consensus.df,
  aes(x = `Factor 1`, y = `Factor 2`, fill = SampleID)) +
  coord_fixed() + xlab("Factor 1") + ylab("Factor 2") +
  geom_point(data = df %>%
               mutate(TableID = factor(TableID)),
             aes(color = SampleID, shape = TableID),
             alpha= 0.8, size  = 3) +
  geom_point(color = "black", size = 5, pch = 23) +
  theme(legend.position = "bottom",
        text = element_text(size = 25)) +
  scale_color_manual(values = cols) +
  scale_fill_manual(values = cols) +
  theme(legend.direction = "horizontal") +
  theme(legend.position = "bottom") +
  theme(legend.box = "vertical")
```

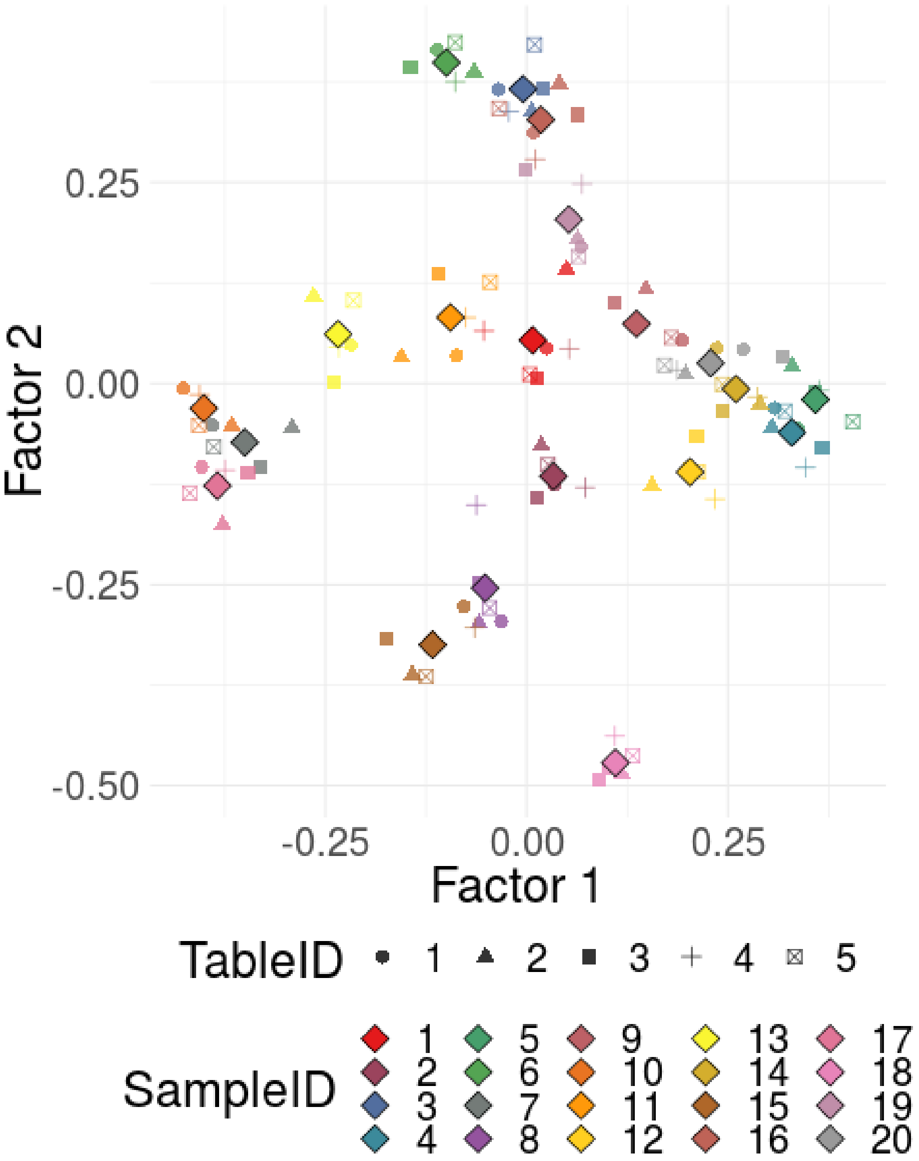

6 Procrustes

Below we generate 20 observations from 2D Gaussian distribution and then project them to 10D (modeling low-rank data embedded in 10D). We then generate 200 bootstrap samples of the data
(200 data subsets with replacement). For each bootstrap trials, we compute the PCA, and save the projections results. We align the results for each bootstrap trial to the original projection of the full dataset.

```
set.seed(123)
n_copies <- 200
n <- 20
p <- 10
```

```
d <- 2
sigma <- 10

X0 <- matrix(rnorm(n*d, sd=sigma), ncol = d)
tmp <- matrix(rnorm(p*p, mean = 0, sd = sigma), ncol = p)
tmp.qr <- qr(tmp)
Q <- qr.Q(tmp.qr, complete=TRUE)
X0 <- X0 %*% t(Q[, seq_len(d)])

res0 <- prcomp(X0)
eigs <- res0$sdev^2/sum(res0$sdev^2)

Y0 <- res0$x[, seq_len(d)]
Y0.df <- data.frame(SampleID = seq_len(n), Y0)

Y.lst <- list()
for(i in seq_len(n_copies)) {
    idx <- unique(sort(sample(seq_len(n), n, replace = TRUE)))
    res <- prcomp(X0[idx, ])
    res.proc <- vegan::procrustes(Y0[idx, ], res$x[, seq_len(d)])
    Y.df <- data.frame(SampleID = idx, res.proc$Yrot)
    Y.lst[[i]] <- Y.df
}
names(Y.lst) <- seq_len(n_copies)
Y.lst.df <- plyr::ldply(Y.lst, .fun = function(x) x, .id = "BootID")
table(Y.lst.df$SampleID)
##
##   1   2   3   4   5   6   7   8   9  10  11  12  13  14  15  16  17  18  19  20
## 139 122 132 125 125 133 128 132 131 137 115 138 124 126 128 123 140 130 126 126
```

Fig. 9(a) showing Procrustes results for 2D "replicated" data (projected to 10D). Density contours are shown for the replicates of the data points.

```
cols <- colorRampPalette(RColorBrewer::brewer.pal(9, "Set1"))(n)
ggplot(
  data = Y.lst.df %>%
      mutate(SampleID = factor(SampleID)),
  aes(x = X1, y = X2, color = SampleID)) +
  coord_fixed() +
  geom_density2d(contour= TRUE) +
  geom_point(alpha= 0.3) +
  geom_point(
      data = Y0.df %>%
      mutate(SampleID = factor(SampleID)),
      aes(fill = SampleID, x = PC1, y = PC2),
      color = "black", size = 5, pch = 23) +
  xlab(paste0("PC 1 [", round(100*eigs[1], 1), "%]")) +
  ylab(paste0("PC 2 [", round(100*eigs[2], 1), "%]")) +
  theme(legend.position = "bottom",
        text = element_text(size = 25)) +
  scale_color_manual(values = cols) +
```

```
      scale_fill_manual(values = cols) +
      xlim(-23, 23) + ylim(-15.5, 15.5)
```

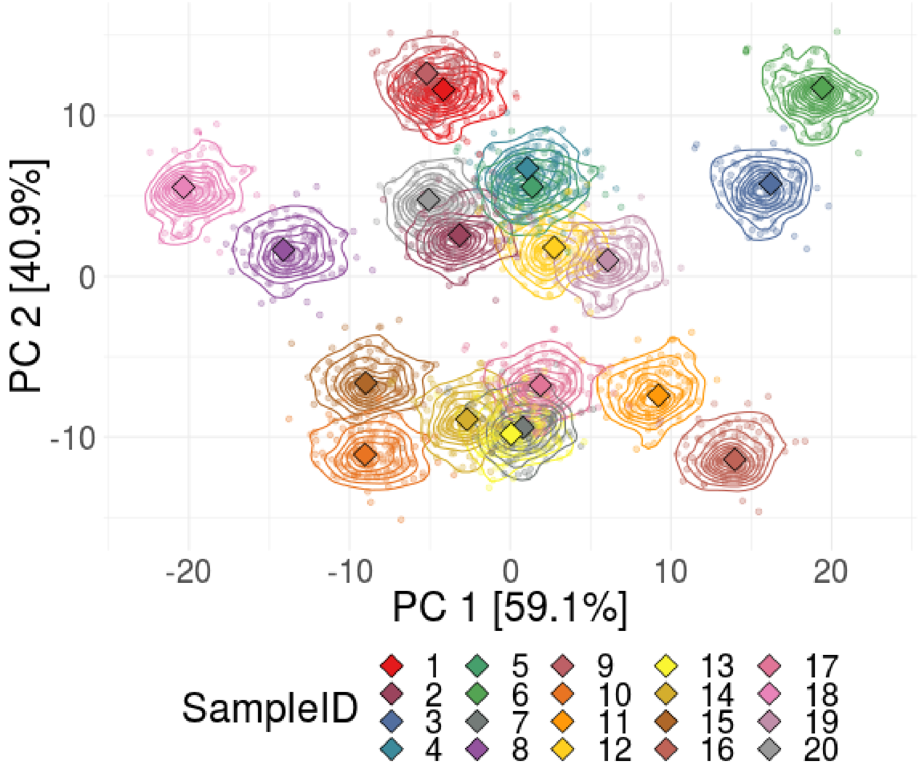

We repeat the process but with 5D Gaussian clusters embedded in 10D.

```
set.seed(1234)
n_copies <- 200
n <- 20
p <- 10
d <- 5
sigma <- 10

X0 <- matrix(rnorm(n*d, sd = sigma), ncol = d)
tmp <- matrix(rnorm(p*p, mean = 0, sd = sigma), ncol = p)
tmp.qr <- qr(tmp)
Q <- qr.Q(tmp.qr, complete=TRUE)
X0 <- X0 %*% t(Q[, seq_len(d)])

res0 <- prcomp(X0)
eigs <- res0$sdev^2/sum(res0$sdev^2)

Y0 <- res0$x[, seq_len(d)]
Y0.df <- data.frame(SampleID = seq_len(n), Y0)

Y.lst <- list()
for(i in seq_len(n_copies)) {
    idx <- unique(sort(sample(seq_len(n), n, replace = TRUE)))
```

```
    res <- prcomp(X0[idx, ])
    res.proc <- vegan::procrustes(Y0[idx, ], res$x[, seq_len(2)])
    Y.df <- data.frame(SampleID = idx, res.proc$Yrot)
    Y.lst[[i]] <- Y.df
}
names(Y.lst) <- seq_len(n_copies)
Y.lst.df <- plyr::ldply(Y.lst, .fun = function(x) x, .id = "BootID")
table(Y.lst.df$SampleID)
##
##   1   2   3   4   5   6   7   8   9  10  11  12  13  14  15  16  17  18  19  20
## 133 109 131 131 125 129 125 129 134 135 133 126 132 138 127 120 133 130 141 124
```

Fig. 9(a) shows the density clusters for the 5D data to be larger, for this dataset.

```
cols <- colorRampPalette(RColorBrewer::brewer.pal(9, "Set1"))(n)
ggplot(
  data = Y.lst.df %>%
      mutate(SampleID = factor(SampleID)),
  aes(x = X1, y = X2, color = SampleID)) +
  coord_fixed() +
  geom_density2d(contour= TRUE) +
  geom_point(alpha= 0.3) +
  geom_point(
      data = Y0.df %>%
      mutate(SampleID = factor(SampleID)),
      aes(fill = SampleID, x = PC1, y = PC2),
      color = "black", size = 5, pch = 23) +
  xlab(paste0("PC 1 [", round(100*eigs[1], 1), "%]")) +
  ylab(paste0("PC 2 [", round(100*eigs[2], 1), "%]")) +
  theme(legend.position = "bottom",
        text = element_text(size = 25)) +
  scale_color_manual(values = cols) +
  scale_fill_manual(values = cols) +
  xlim(-31, 31) + ylim(-28, 28)
```

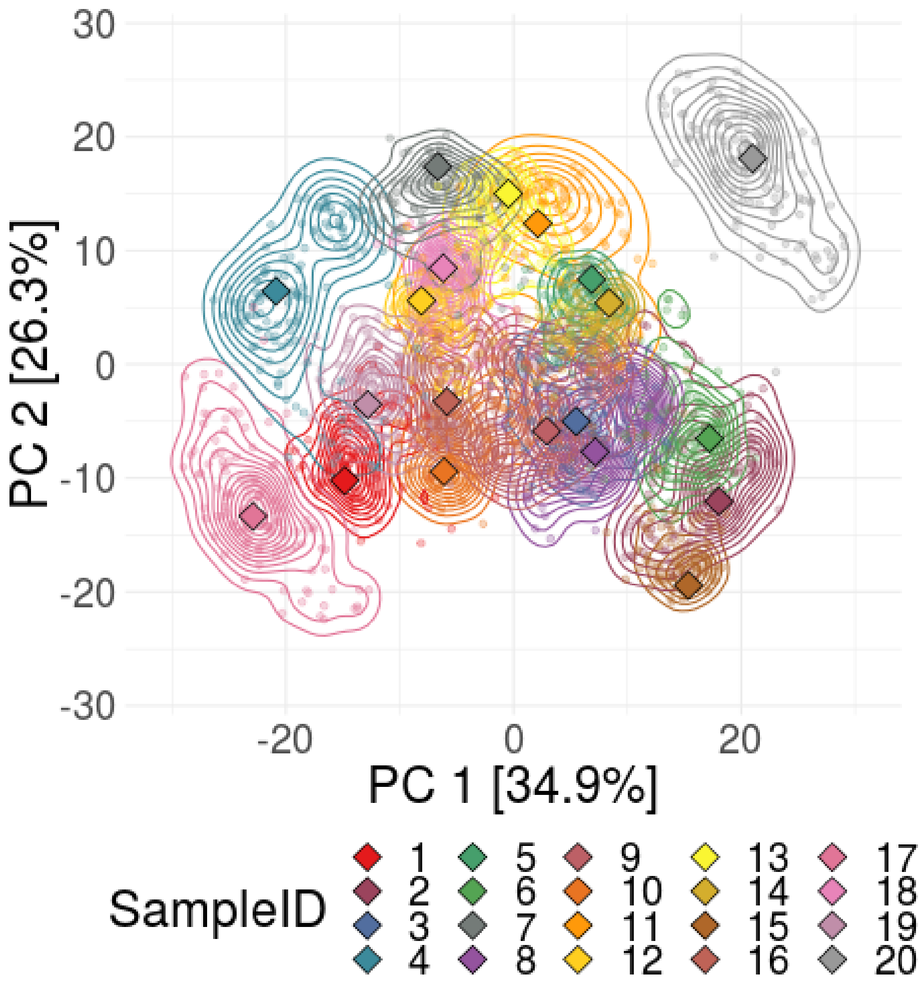

7 Eigenvalues instability

We simulate the data with very close eigenvalues:

```
set.seed(4756)
n <- 100
p <- 10
R1 <- matrix(rnorm(n*p), nrow = n)
R2 <- matrix(rnorm(p*p), nrow = p)
U <- qr.Q(qr(R1))
V <- qr.Q(qr(R2))
eigs <- c(7, 5.3, 5.2, 5.1, 3.5, 3, 2.5, 2*runif(3))
X <- U %*% diag(eigs) %*% t(V)
```

Fig. 8 showing a scree plot with similar eigenvalues indicating unstable PCs.

```
pca.res <- prcomp(X)
fviz_eig(pca.res) +
  ggtitle("") +
```

```
  theme(text = element_text(size = 25))
```

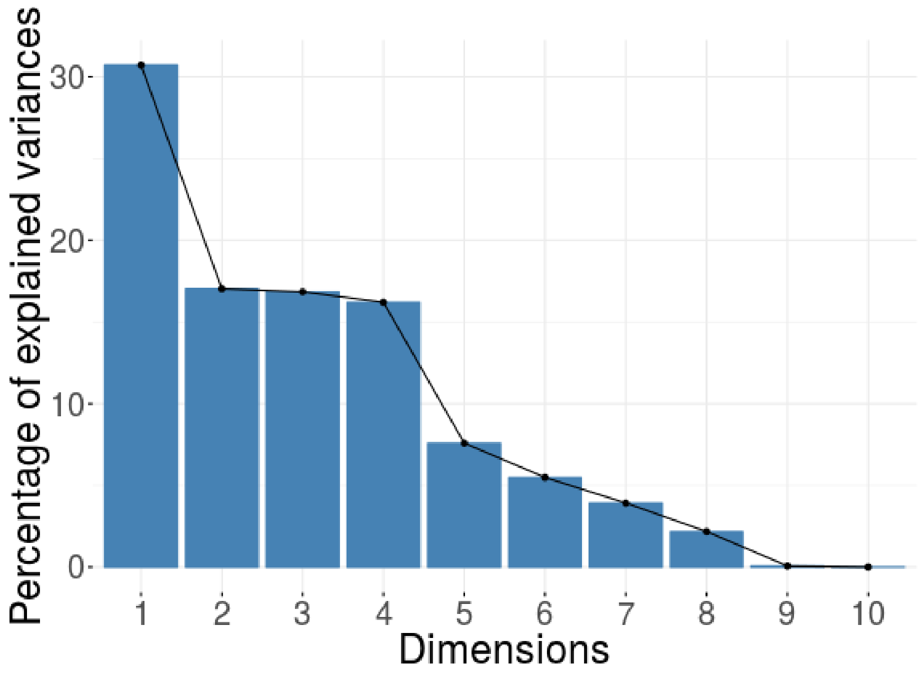


```
fviz_pca_ind(pca.res) +   theme(text = element_text(size = 25))
```

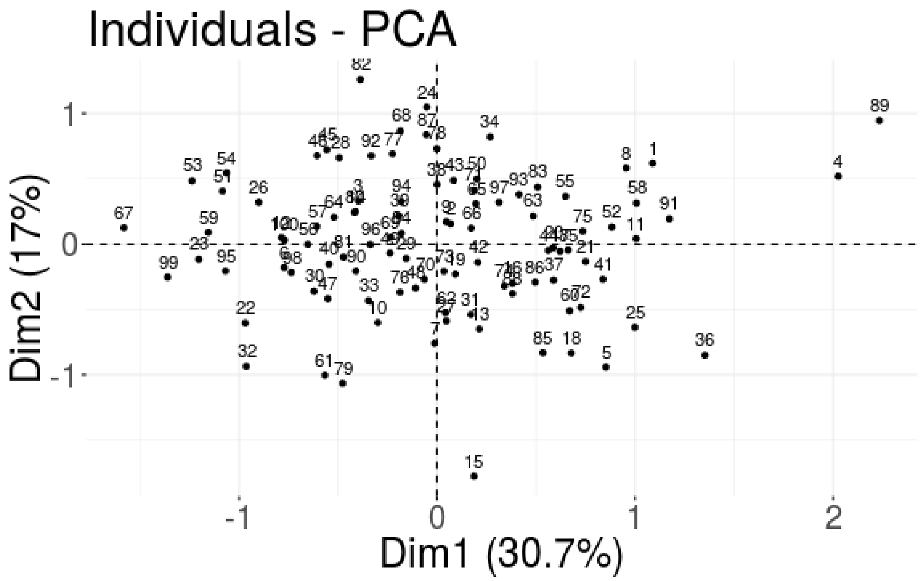


```
sigma <- 0.1
E <- matrix(sigma*rnorm(n*p), ncol = p)
pca.res.perturbed <- prcomp(X + E)
fviz_eig(pca.res.perturbed) +
  theme(text = element_text(size = 25))
```

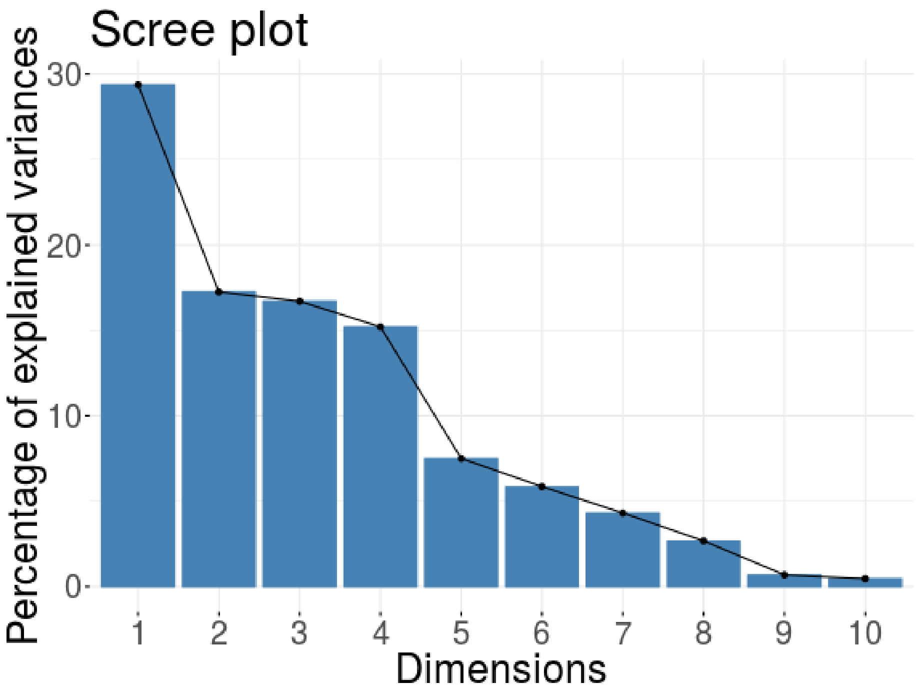

PCA results changed quite a bit with a small perturbation

```r
fviz_pca_ind(pca.res.perturbed)
```

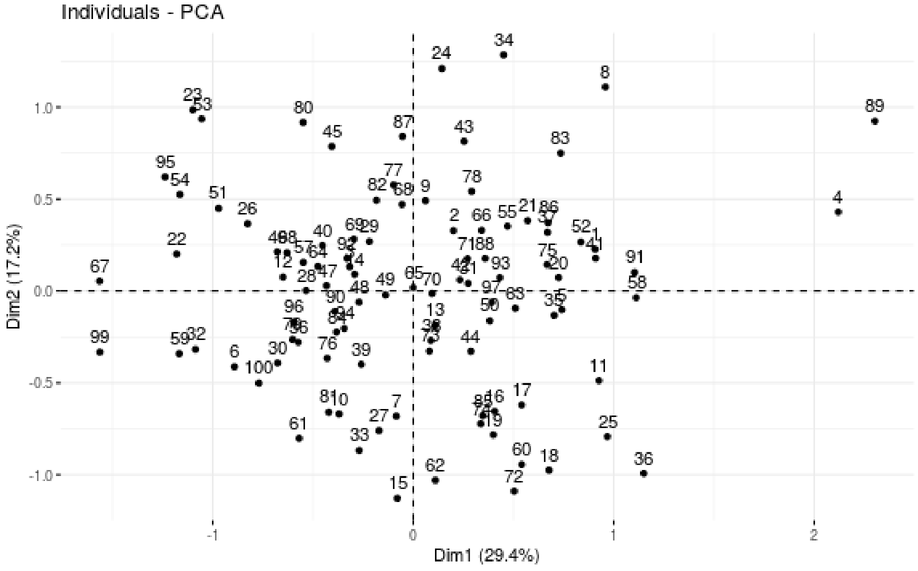

8 Striking image

GP data

```r
set.seed(8567)
n <- 100
```

```
p <- 3
sigma <- 0.01
t <- sort(runif(n))
D <- as.matrix(dist(t))
K <- exp(-2*(D^2)) + sigma*diag(n)
eigen.res <- eigen(K)
ev <- pmax(eigen.res$values, 0)
A <- eigen.res$vectors %*% diag(sqrt(ev))
Z <- matrix(rnorm(n*p), ncol = p)
M <- A %*% Z
M <- data.frame(
  x = MASS::mvrnorm(mu = rep(0, times = n), Sigma = K),
  y = MASS::mvrnorm(mu = rep(0, times = n), Sigma = K),
  z = MASS::mvrnorm(mu = rep(0, times = n), Sigma = K))

pca.res <- prcomp(M)

X <- data.frame(pca.res$x)
colnames(X)[1:3] <- c("x", "y", "z")
X$color <- t
```

```
theme.novpadding <-
  list(layout.heights =
         list(top.padding = 0,
              main.key.padding = 0,
              key.axis.padding = 0,
              axis.xlab.padding = 0,
              xlab.key.padding = 0,
              key.sub.padding = 0,
              bottom.padding = 0),
       axis.line = list(col = 0),
       clip =list(panel="off"),
       layout.widths =
         list(left.padding = 0,
              key.ylab.padding = 0,
              ylab.axis.padding = 0,
              axis.key.padding = 0,
              right.padding = 0))

classic_theme <- theme_classic() +
    theme(
        axis.text.x = element_blank(),
        axis.ticks.x = element_blank(),
        axis.text.y = element_blank(),
        axis.ticks.y = element_blank())
```

```
M$color <- t
p1 <- ggplot(X) +
  geom_point(aes(color, 0, color = color)) + #, pch = 21, size = 3, color = "white") +
  coord_fixed() + ylab("") + xlab("x") +
```

```
      classic_theme + theme(axis.line.y =element_blank()) +
      scale_color_viridis_c(guide = "none") +
      scale_fill_viridis_c(guide = "none") +
      theme(plot.margin = unit(c(0,0.5,0,0), "cm"))
    p2 <- ggplot(X) +
        geom_point(aes(x, y, color = color)) +
        coord_fixed() +
        scale_color_viridis_c(guide = "none") +
        classic_theme +
        theme(plot.margin = unit(c(0,0.5,0,0), "cm"))

    p3 <- lattice::cloud(
      z ~ x*y, M, pch = 19,
      col.point=viridis(length(X$color))[order(X$color)],
      par.settings = theme.novpadding)
```

```
grid.arrange(p3, p2, p1, ncol = 2,
             layout_matrix = cbind(c(1,1,1, 3), c(2, 2, 2, 3)),
             widths = c(0.5, 0.5),
             padding = unit(0.00, "line"))
```

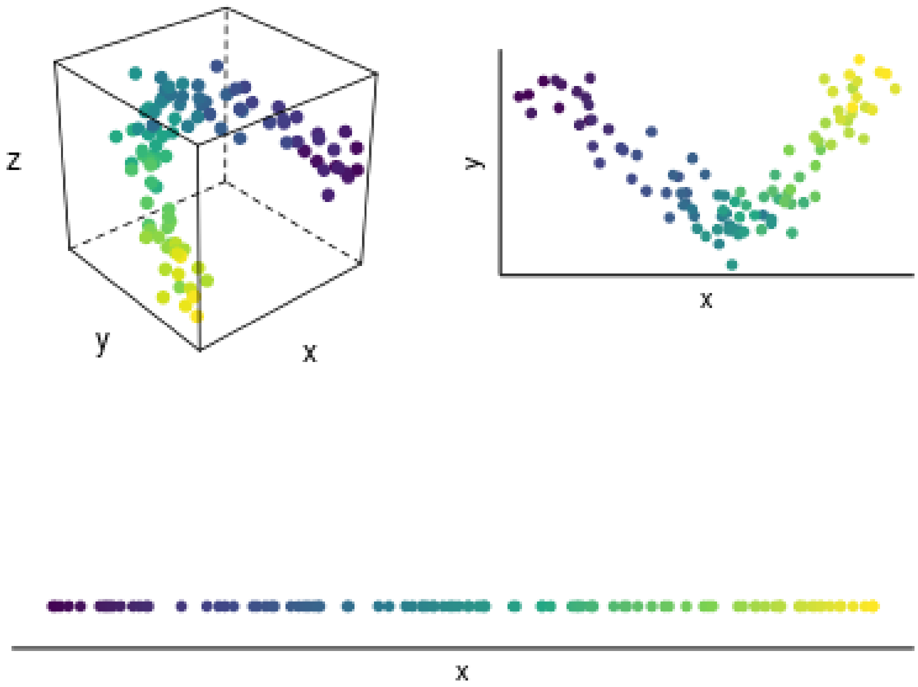


```
sessionInfo()
## R version 3.5.1 (2018-07-02)
## Platform: x86_64-pc-linux-gnu (64-bit)
## Running under: Ubuntu 16.04.5 LTS
##
## Matrix products: default
## BLAS: /usr/lib/libblas/libblas.so.3.6.0
## LAPACK: /usr/lib/lapack/liblapack.so.3.6.0
```

```
##
## locale:
##  [1] LC_CTYPE=en_US.UTF-8       LC_NUMERIC=C               LC_TIME=en_US.UTF-8        LC_COLLATE=en_US.UTF
##  [8] LC_NAME=C                  LC_ADDRESS=C               LC_TELEPHONE=C             LC_MEASUREMENT=en_US
##
## attached base packages:
## [1] stats     graphics  grDevices utils     datasets  methods   base
##
## other attached packages:
##  [1] ade4_1.7-13       bindrcpp_0.2.2    RMTstat_0.3       viridis_0.5.1     viridisLite_0.3.0 gridExtra_2
## [11] readr_1.2.1       tidyr_0.8.2       tibble_1.4.2      tidyverse_1.2.1   lattice_0.20-38   factoextra_
##
## loaded via a namespace (and not attached):
##  [1] ggrepel_0.8.0      Rcpp_1.0.0         lubridate_1.7.4    assertthat_0.2.0   rprojroot_1.3-2    digest
## [11] backports_1.1.2    evaluate_0.12      httr_1.3.1         pillar_1.3.1       rlang_0.3.0.1      lazyev
## [21] Matrix_1.2-15      labeling_0.3       munsell_0.5.0      broom_0.5.0        compiler_3.5.1     modelr
## [31] tidyselect_0.2.5   bookdown_0.7       permute_0.9-4      fansi_0.4.0        crayon_1.3.4       withr_
## [41] jsonlite_1.6       gtable_0.2.0       magrittr_1.5       scales_1.0.0       cli_1.0.1          string
## [51] glue_1.3.0         hms_0.4.2          parallel_3.5.1     abind_1.4-5        yaml_2.2.0         colors
## [61] knitr_1.20         bindr_0.1.1        haven_2.0.0
```
